# Enteric α-synuclein impairs intestinal epithelial barrier through caspase-1-inflammasome signaling in Parkinson’s disease before brain pathology

**DOI:** 10.1038/s41531-021-00263-x

**Published:** 2022-01-12

**Authors:** C. Pellegrini, V. D’Antongiovanni, F. Miraglia, L. Rota, L. Benvenuti, C. Di Salvo, G. Testa, S. Capsoni, G. Carta, L. Antonioli, A. Cattaneo, C. Blandizzi, E. Colla, M. Fornai

**Affiliations:** 1 Unit of Histology and Medical Embryology, Department of Clinical and Experimental Medicine, University of Pisa, Pisa, Italy; 2 Unit of Pharmacology and Pharmacovigilance, Department of Clinical and Experimental Medicine, University of Pisa, Pisa, Italy; 3 Bio@SNS Laboratory, Scuola Normale Superiore, Pisa, Italy; 4 Department of Biomedical Science, University of Cagliari, Cagliari, Italy

**Keywords:** Cellular neuroscience, Parkinson's disease

## Abstract

Bowel inflammation, impaired intestinal epithelial barrier (IEB), and gut dysbiosis could represent early events in Parkinson’s disease (PD). This study examined, in a descriptive manner, the correlation among enteric α-synuclein, bowel inflammation, impairments of IEB and alterations of enteric bacteria in a transgenic (Tg) model of PD before brain pathology. Human A53T α-synuclein Tg mice were sacrificed at 3, 6, and 9 months of age to evaluate concomitance of enteric inflammation, IEB impairments, and enteric bacterial metabolite alterations during the early phases of α-synucleinopathy. The molecular mechanisms underlying the interplay between α-synuclein, activation of immune/inflammatory responses and IEB alterations were investigated with in vitro experiments in cell cultures. Tg mice displayed an increase in colonic levels of IL-1β, TNF, caspase-1 activity and enteric glia activation since 3 months of age. Colonic TLR-2 and zonulin-1 expression were altered in Tg mice as compared with controls. Lipopolysaccharide levels were increased in Tg animals at 3 months, while fecal butyrate and propionate levels were decreased. Co-treatment with lipopolysaccharide and α-synuclein promoted IL-1β release in the supernatant of THP-1 cells. When applied to Caco-2 cells, the THP-1-derived supernatant decreased zonulin-1 and occludin expression. Such an effect was abrogated when THP-1 cells were incubated with YVAD (caspase-1 inhibitor) or when Caco-2 were incubated with anakinra, while butyrate incubation did not prevent such decrease. Taken together, early enteric α-synuclein accumulation contributes to compromise IEB through the direct activation of canonical caspase-1-dependent inflammasome signaling. These changes could contribute both to bowel symptoms as well as central pathology.

## Introduction

Gastrointestinal (GI) dysfunctions, including dysphagia, infrequent bowel movements, and constipation, are among the most common non-motor symptoms of Parkinson’s disease (PD)^1–3^. Such GI disturbances can occur since the earliest stages of PD and represent prodromal events of the disease^4^. In this context, growing evidence support the contention that enteric α-synuclein (α-syn) inclusions (a hallmark of PD), changes in gut microbiota composition, impaired intestinal epithelial barrier (IEB), and bowel inflammation could contribute to bowel motor dysfunctions as well as to neuroinflammation and neurodegeneration in the central nervous system (CNS)^4–6^. Indeed, both human and pre-clinical studies have reported that PD is associated with alterations of enteric bacteria and their metabolites, impaired IEB, enteric accumulation of aggregated and phosphorylated α-syn and gut inflammation^6–8^.

In a recent paper, we have demonstrated that A53T α-syn mice (a genetic model of α-synucleinopathy, reflecting one of the major features of PD) displayed an accumulation of insoluble and aggregated α-syn in enteric neurons in both myenteric and submucosal plexi along with colonic motor abnormalities, characterized by an impairment of cholinergic neurotransmission in the early stages of α-syn-driven pathology without concomitant CNS involvement^9^. However, a clear causal relationship between alterations of enteric bacteria, altered IEB, enteric α-syn accumulation, bowel inflammation, and PD pathology remains still unclear.

Based on the above background, the present study was designed to address two relevant issues: (1) the concomitance of altered gut microbiota metabolites, enteric α-syn accumulation, impaired IEB and bowel inflammation in a transgenic model of PD (A53T α-syn Tg), before the onset of neurodegeneration in the CNS and (2) the correlation among α-syn accumulation, immune/inflammatory responses, and alterations of IEB. This knowledge provides new insights to a better understanding of the mechanisms underlying intestinal dysfunctions in PD as well as identifying rational therapeutic approaches for their management.

## Results

### Enteric inflammatory responses in pre-symptomatic transgenic (Tg) mice precede brain inflammation

**Fig. 1:**
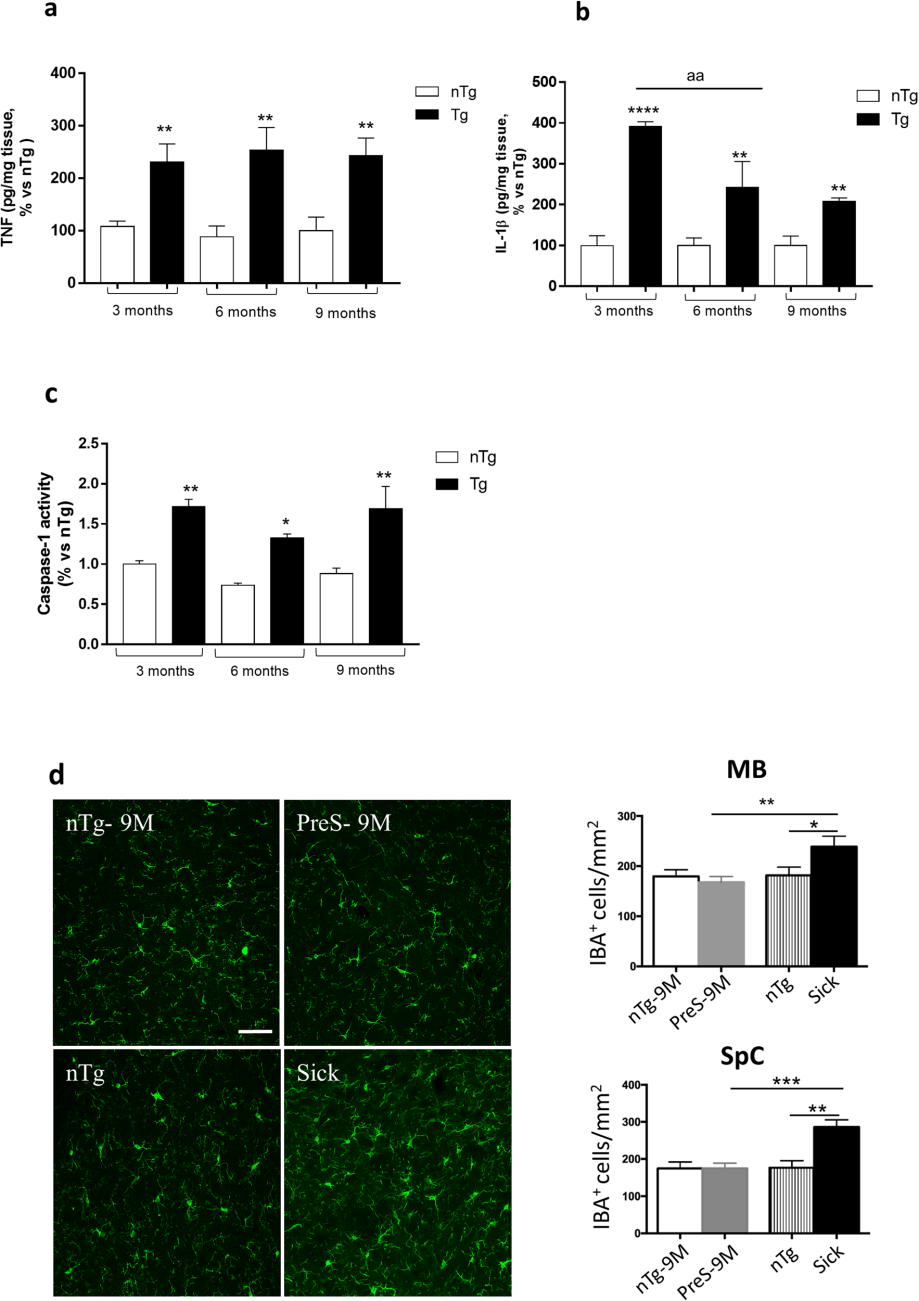
Enteric inflammatory responses in pre-symptomatic Tg mice precede brain inflammation. **a** TNF levels in colonic tissues from nTg and Tg mice at 3, 6, and 9 months of age. Values represent the means ± S.E.M. One-way ANOVA followed by Tukey post hoc test results: **P < 0.01, significant difference versus nTg age-matched controls; **b** IL-1β levels in colonic tissues from nTg and Tg mice at 3, 6, and 9 months of age. Values represent the means ± SEM. One-way ANOVA followed by Tukey post hoc test results: **P < 0.01, ****P < 0.001 significant difference versus nTg age-matched controls, and ^aa^*P* < 0.01 significant difference; **c** Caspase-1 activity in colonic tissues from nTg and Tg mice at 3, 6, and 9 months of age. Values represent the means ± SEM. One-way ANOVA followed by Tukey post hoc test results: *P < 0.05 and **P < 0.01, significant difference versus nTg age-matched controls); **d** Confocal analysis of IBA-1 positive cells in MB and SpC of presymptomatic (9 months), sick and nTg mice. Images were acquired using a 40× objective of Leica TCS SP confocal laser-scanning microscope and cell density analysis was obtained using ImageJ. Values represent the means ± SEM. One-way ANOVA followed by Fisher post hoc test results: *P < 0.05; **P < 0.01; ***P < 0.001 significant difference. Scale bar, 100 nm. *n* = 3–5/group. *IL-1β* interleukin-1beta, MB midbrain, SpC spinal cord, *nTg* non-transgenic, *Tg* transgenic, *TNF* tumor necrosis factor.

In order to verify whether intestinal inflammation represents an early event in PD before the onset of central neuroinflammatory and neurodegenerative processes, we evaluated colonic pro-inflammatory interleukin (IL)-1β and tumor necrosis factor (TNF) levels in pre-symptomatic Tg mice at 3, 6, and 9 months of age. The results showed that Tg mice displayed a significant increase in both cytokine levels starting from 3 months of age, as compared with their control littermates (nTg) (Fig. 1a, b). Of note, since the release of mature IL-1β can result from the cleavage of several caspases, including caspase-1^10,11^, we investigated caspase-1 activity in colonic tissues from Tg mice. Interestingly, we found that caspase-1 activity was significantly increased in colonic tissues from Tg mice at 3, 6, and 9 months of age, as compared with nTg animals (Fig. 1c), indicating the activation of the canonical caspase-1 dependent inflammasome signaling. In addition, an increased GFAP-positive glial cells in the mucosa, submucosa and myenteric plexus were observed in Tg mice at 3 months of age, as compared with nTg animals, thus suggesting enteric glia activation (Supplementary Fig. 1). These results suggest that peripheral immune/inflammatory responses represent an early event in PD before the onset of brain pathology. Confirming these results, we examined the activation of microglia in the CNS of this Tg line, since neurodegeneration, which appears after 9 months of age, is often associated with neuroinflammation^12^. In particular, we analyzed the density of IBA-1, a biochemical marker of glia activation, in the spinal cord and midbrain tissues, two areas largely affected by neurodegeneration in adult α-syn Tg mice. Of note, no changes in IBA-1 density were observed in pre-symptomatic and 9 months old Tg animals, as compared with age-matched controls (Fig. 1d), whereas a significant and expected increase in IBA-1 density was detected in the midbrain and spinal cord tissues from sick Tg mice (Fig. 1d).

### Pre-symptomatic Tg mice display impairments of IEB integrity and permeability

The occurrence of enteric inflammation has been found to be related to an impairment of the enteric epithelial barrier with a consequent increase in intestinal permeability^13^. Therefore, we went on to examine colonic expression of tight junction proteins, zonulin-1 (ZO-1) and occludin as well as circulating lipopolysaccharide (LPS) levels, regarded as an indirect index of intestinal permeability^14^ in pre-symptomatic Tg mice.

**Fig. 2:**
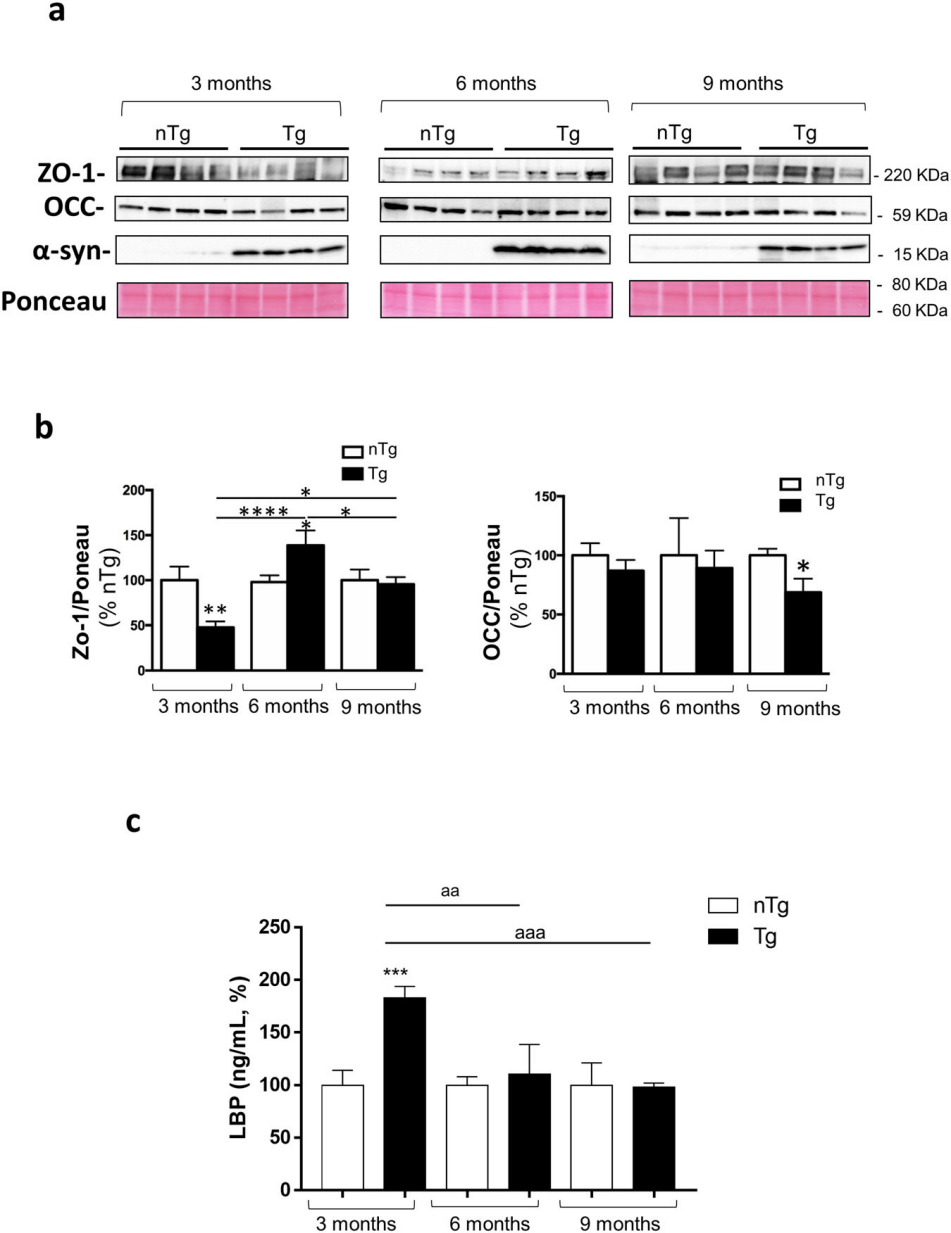
Pre-symptomatic Tg mice display impairments of IEB integrity and permeability. **a** Representative blots and (**b**) densitometric analysis of ZO-1 and Occ expression assessed by Western blot assay in colonic tissues from nTg and Tg mice at 3, 6, and 9 months of age. In the quantitative analysis protein levels were normalized using Ponceau staining and expressed as percentage of the mean of nTg age-matched controls. All values represent the means ± SEM. *P < 0.05, **P < 0.01, ****P < 0.0001, significant difference. One-way ANOVA followed by Tukey post hoc test. **c** Circulating LBP in nTg and Tg mice at 3, 6, and 9 months of age. All values represent the means ± SEM. ***P < 0.001, significant difference versus nTg age-matched controls), ^aaa^*P* < 0.001 and ^aa^*P* < 0.01. One-way ANOVA followed by Tukey post hoc test. *n* = 3–5/group. α-S α-synuclein, *LBP* lipopolysaccharide-binding protein, *nTg* non-transgenic, *Tg* transgenic, Occ occludin, *ZO-1* zonulin-1.

Significant decrease in colonic ZO-1 expression was found in Tg mice already at 3 months of age, while it increased at 6 months and normalized at 9 months. With regard for the occludin, we found a decrease in this tight junction protein expression in colonic tissues from 9 months old Tg mice, as compared with nTg littermates (Fig. 2a, b). As such alterations can impair IEB integrity with consequent increase in gut permeability^15^, we analyzed plasma levels of LPS in pre-symptomatic Tg mice and found a significant increase in Tg mice at 3 months of age, while no differences were observed in Tg mice at 6 and 9 months as compared with nTg age-matched controls (Fig. 2c).

### Pre-symptomatic Tg mice display an altered toll-like receptor (TLR)-2 expression in colonic tissues

**Fig. 3:**
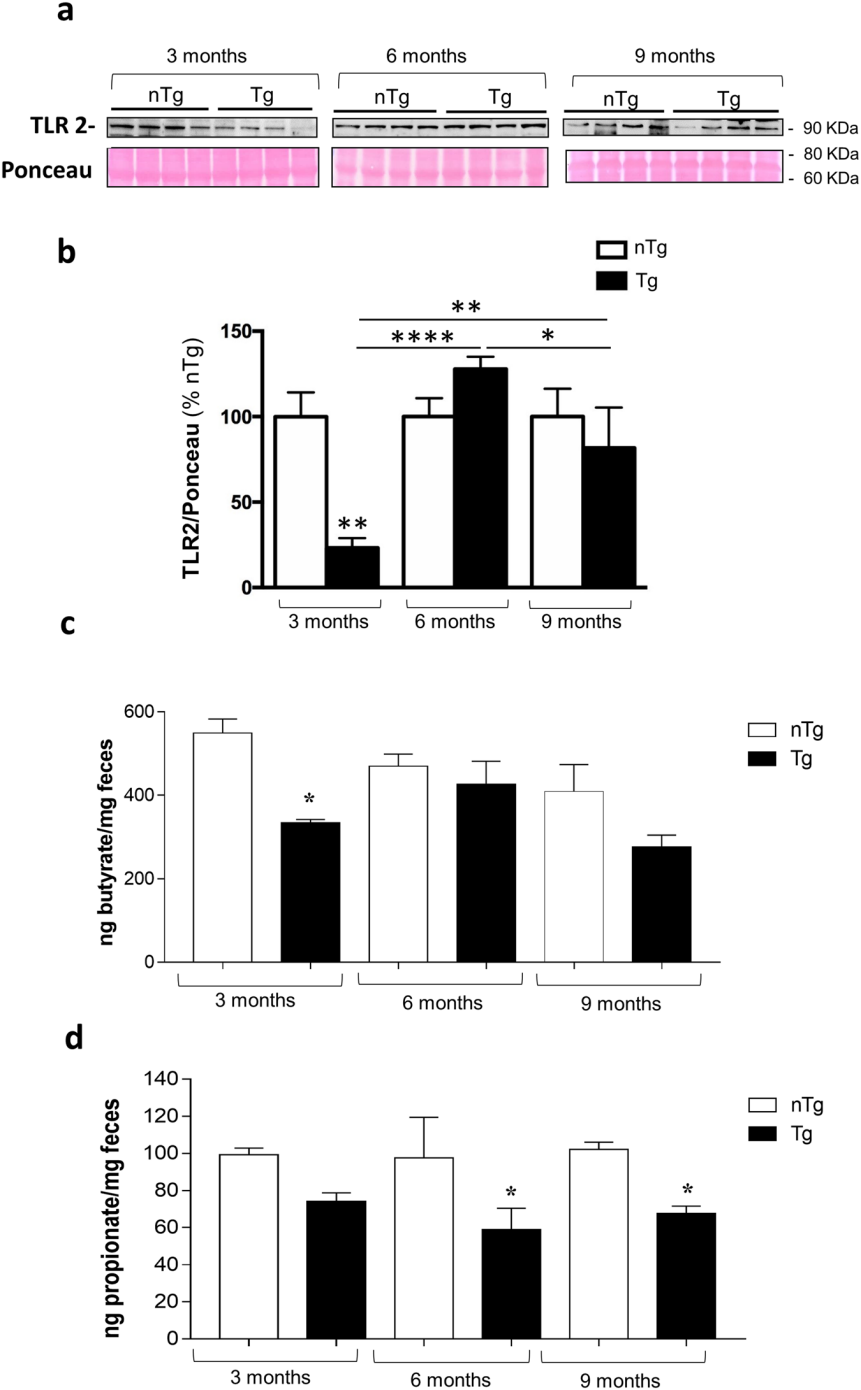
Pre-symptomatic Tg mice display an altered TLR-2 expression in colonic tissues and a decrease in fecal butyrate and propionate levels since 3 months of age. **a** Representative blots and (**b**) densitometric analysis of TLR-2 expression assessed by Western blot assay in colonic tissues from nTg and Tg mice at 3, 6, and 9 months of age. In the quantitative analysis protein levels were normalized using Ponceau staining and expressed as percentage of the mean of nTg age-matched controls. All values represent the mean ± SEM. *P < 0.05, **P < 0.01, ****P < 0.0001, significant difference. One-way ANOVA followed by Tukey post hoc test. **c** Butyrate and (**d**) propionate levels in the feces from nTg and Tg mice at 3, 6, and 9 months of age. All values represent the means ± SEM. Two-way ANOVA followed by Tukey post hoc test results: *P < 0.05, significant difference versus nTg age-matched controls). *n* = 3–5/group. *nTg* non-transgenic, *SCFAs* fermentation products of enteric bacteria, *Tg* transgenic, *TLR-2* toll like receptor-2.

In order to better understand the mechanisms underlying the occurrence of enteric inflammation and impairment of IEB in early PD, we examined the expression of TLR-2 in colonic tissues from pre-symptomatic Tg mice. This receptor subtype, expressed on neuronal, immune/inflammatory and intestinal epithelial cells, is involved in the occurrence of inflammatory responses, maintenance of the IEB integrity as well as shaping gut microbiota^16,17^. Our results showed that Tg mice displayed a decrease in colonic TLR-2 expression at 3 months of age, an increase at 6 months while no significant alterations were observed at 9 months of age as compared with nTg littermates (Fig. 3a, b).

In addition, an increased co-localization of GFAP-positive glial cells (red fluorescence) with TLR-2 (green fluorescence, white arrows) was observed in Tg mice at 3 months of age, as compared with nTg mice (Supplementary Fig. 1).

### Pre-symptomatic Tg mice display a decrease in fecal butyrate and propionate levels since 3 months of age

Alterations of short-chain fatty acid (SCFA) levels, related to the occurrence of gut dysbiosis, are often associated with enteric inflammation and alterations of IEB during intestinal inflammatory conditions^13^. Therefore, we assessed butyrate and propionate levels in the feces of Tg mice. Our results showed a significant decrease in butyrate levels in Tg mice at 3 months of age, as compared with nTg animals, while no changes in butyrate concentration were observed in older mice at 6 and 9 months of age (Fig. 3c). With regard for propionate levels, significant decrease was observed in pre-symptomatic Tg mice at 6 and 9 months of age as compared with nTg animals (Fig. 3d). These results suggest a decrease in fecal SCFA levels in early Tg mice before the onset of CNS pathology, indicating alterations of enteric bacteria functions.

### Enteric α-syn triggers the release of IL-1β from activated macrophage, inducing an increase in intestinal epithelial tight junction permeability

In order to verify whether α-syn can promote macrophage activation, we performed in vitro experiments in the LPS-primed phorbol 12-myristate 13-acetate (PMA)-differentiated THP-1 cell line, a well-established model to study immune/inflammatory cell activation, mechanisms and signaling pathways^18^. In particular, we tested the ability of soluble full length of α-syn (including monomers), regarded as one of the α-syn species most expressed in colonic tissues from Tg mice since 3 month old^9^, of inducing the release of IL-1β, a key mediator of inflammatory response^19–21^.

**Fig. 4:**
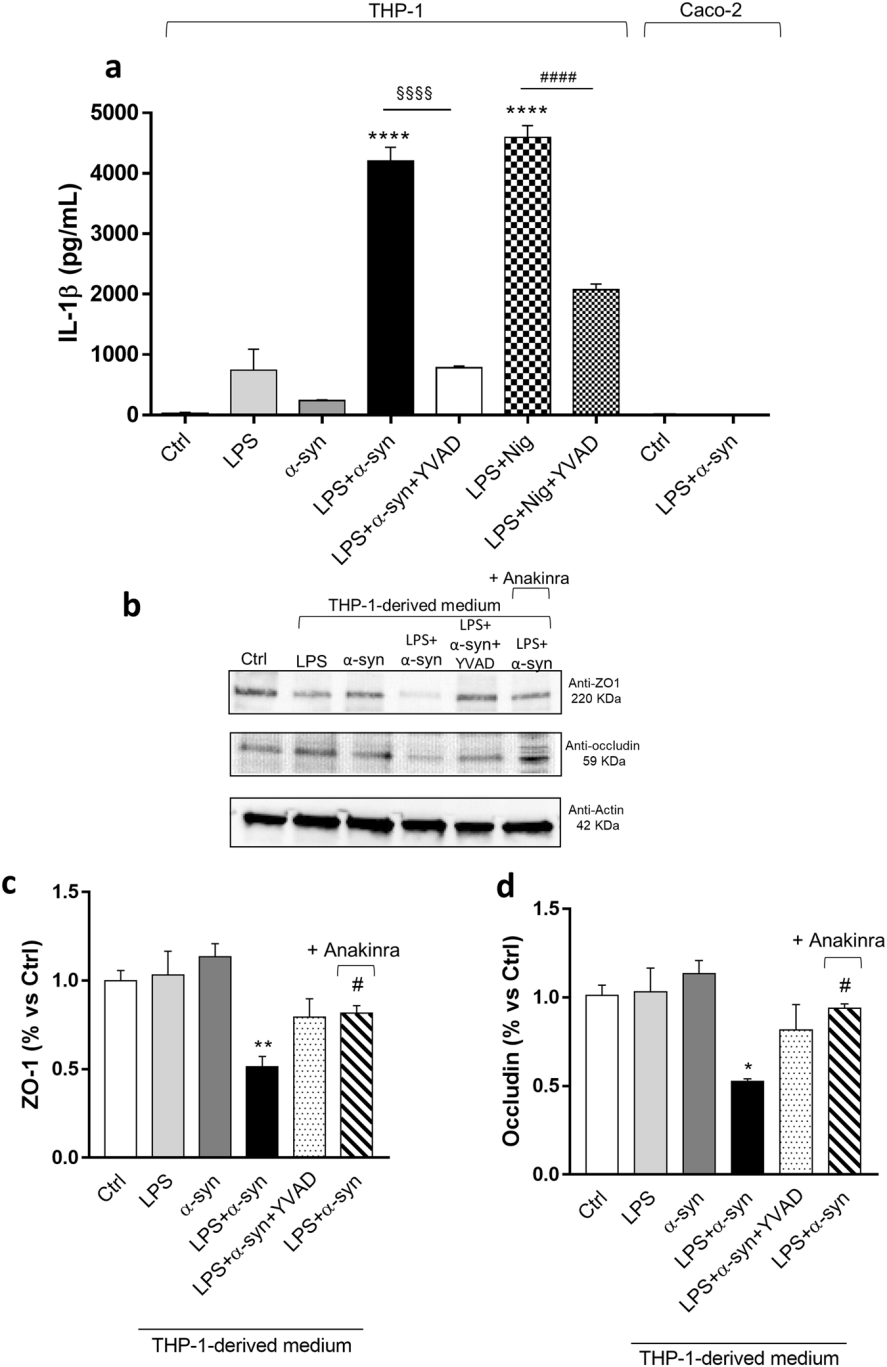
Enteric accumulation of α-syn triggers the release of IL-1β from activated macrophage, which, in turn, induces an increase in intestinal epithelial tight junction permeability. **a** IL-1β levels in the supernatants of THP-1 cells treated with LPS alone or in combination with α-syn or Nig, in the presence or in the absence of YVAD. IL-1β levels in the supernatants of Caco-2 cells untreated or treated with LPS plus α-syn. All values represent the mean ± SEM. One-way ANOVA followed by Tukey post hoc test results: ****P < 0.0001 significant difference versus LPS-primed THP-1 (Ctrl); ^§§§§^*P* < 0.0001, significant difference versus LPS-primed THP-1 treated with α-syn plus LPS; ^####^*P* < 0.0001, significant difference versus LPS-primed THP-1 treated with LPS plus Nig; **b** Representative blots and densitometric analysis of c ZO-1 and (**d**) occludin expression assessed by Western blot assay in cultured Caco-2 cells treated with conditioned medium derived from THP-1 cells. All values represent the mean ± SEM. One-way ANOVA followed by Tukey post hoc test results: *P < 0.05, **P < 0.01 significant difference versus Caco-2 cells (Ctrl); ^#^*P* < 0.05 significant difference versus Caco-2 cells treated with α-syn plus LPS. *n* = 5 independent experiments. *α-syn* α-synuclein, *IL-1β* interleukin-1beta, *LPS* lipopolysaccharide, *Nig* nigericin, *ZO-1* zonulin-1.

In THP-1 cells, the incubation of α-syn did not induce a significant release of IL-1β (Fig. 4a). In LPS-primed THP-1 cells, treatment with α-syn or nigericin (Nig, positive control) induced a massive release of IL-1β (Fig. 4a). Interestingly, treatment with caspase-1 inhibitor, YVAD, reduced significantly the release of IL-1β induced by α-syn or Nig in the presence of LPS (Fig. 4a), suggesting that α-syn can promote the release of IL-1β through the activation of the canonical caspase-1-dependent inflammasome complex in immune/inflammatory cells.

As the increase in IL-1β levels can impair IEB integrity and permeability^22,23^, we investigated the effects of the IL-1β release from THP-1 cells on the expression levels of tight junction proteins in human epithelial Caco-2 cell line. To pursue this goal, Caco-2 cells were treated with the conditioned medium derived from activated macrophages, (previously treated with α-syn alone or in combination of LPS, in absence or in the presence of caspase-1 inhibitor, YVAD) and IL-1β receptor antagonist, anakinra.

**Fig. 5:**
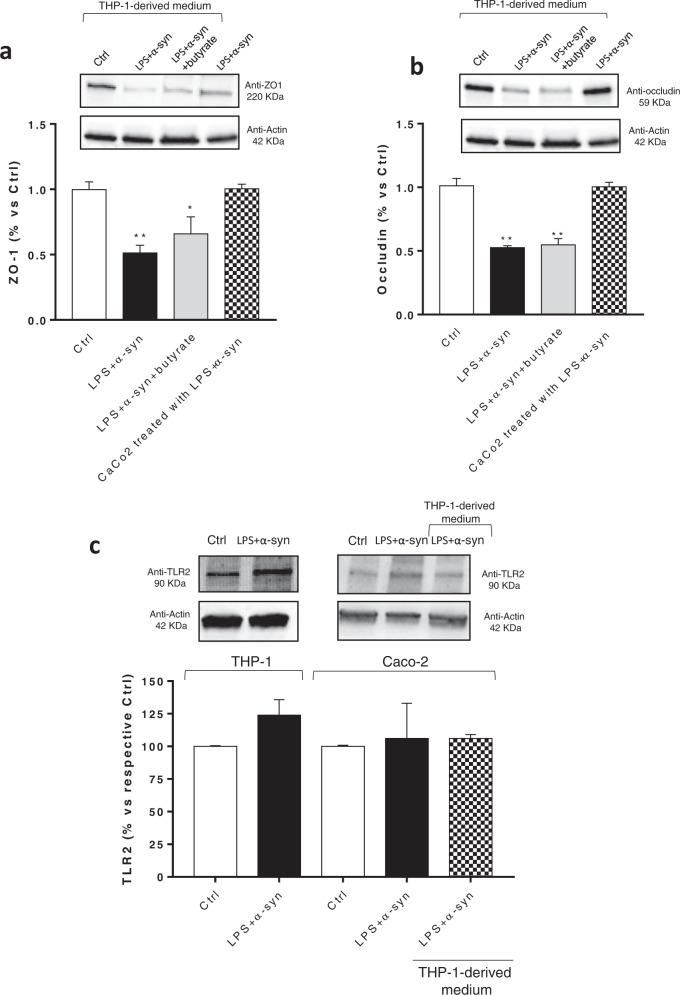
Incubation of α-syn plus LPS directly in the medium of Caco-2 cells does not alter the expression ZO-1 and occludin. **a, b** Representative blots and densitometric analysis of ZO-1 and occludin expression assessed by Western blot assay in cultured Caco-2 cells treated with conditioned medium derived from THP-1 cells treated with α-syn plus LPS, in the absence or in the presence of butyrate, or with α-syn plus LPS directly in the medium. All values represent the mean± SEM. One-way ANOVA followed by Tukey’s post hoc test results: *P < 0.05, **P < 0.01 significant difference versus Caco-2 cells (Ctrl). **c** Representative blots and densitometric analysis of TLR-2 expression assessed by Western blot assay in cultured THP-1 and Caco-2 cells treated with LPS and α-syn and Caco-2 incubated with conditioned medium derived from THP-1 cells treated with α-syn and LPS. All values represent the mean ± SEM. Student’s *t* test and one-way ANOVA followed by Tukey’s post hoc test results: no significant difference versus respective Ctrl. *n* = 5 independent experiments. *α-syn* α-synuclein, *LPS* lipopolysaccharide, TLR-2 toll like receptor-2, *ZO-1* zonulin-1.

In Caco-2 cells, the incubation with conditioned medium derived from THP-1 cells treated with LPS or α-syn alone did not alter the expression of ZO-1 and occludin (Fig. 4b-d). By contrast, treatment with conditioned medium derived from THP-1 cells treated with α-syn plus LPS significantly reduced the expression of ZO-1 and occludin, as compared with control cells (Fig. 4b-d). Such effect was counteracted when Caco-2 cells were treated with the conditioned medium derived from THP-1 cells, previously incubated with caspase-1 inhibitor, YVAD as well as when Caco-2 cells were treated with IL-1β receptor antagonist, anakinra (Fig. 4b-d). Of note, the incubation of α-syn plus LPS in the medium of Caco-2 cells did not alter the expression of ZO-1 and occludin, indicating that these mediators did not produce any direct effect on tight junction protein expression in intestinal epithelial cells (Fig. 5a, b). The incubation with butyrate did not prevent the reduction of ZO-1 and occludin expression in Caco-2 cells treated with the conditioned medium derived from THP-1 cells (Fig. 5a, b).

Of note, direct incubation of LPS and α-syn with THP-1 and Caco-2 cells, as well as treatment of Caco-2 cells with conditioned medium derived from THP-1 cells treated with α-syn plus LPS did not affect TLR-2 expression (Fig. 5c).

## Discussion

Growing evidence support the contention that gut dysbiosis and changes in their metabolite production (i.e., SCFAs), impairment of IEB, enteric α-syn inclusions and bowel inflammation represent earliest events in PD that could contribute to intestinal motor dysfunctions as well as brain pathology via gut-brain ascending pathways^4,6^. However, the relationship among enteric α-syn accumulation, alterations of enteric bacteria-immune network, and PD pathology remains to be clarified.

On these bases, the purpose of the present study was to examine the correlation among enteric α-syn accumulation, bowel inflammation, alterations of IEB and changes in gut SCFA in a transgenic model of PD before the onset of brain pathology. In this setting, the A53T α-syn Tg mouse can be an extremely valuable experimental model to investigate the intestinal symptoms in the prodromal phases of PD^9,24^.

Taken together, the results of the present study show that, in the early stages of PD, enteric α-syn accumulation can compromise IEB through direct activation of innate immune/inflammatory cells, providing evidence about a direct involvement of α-syn in the alterations of enteric bacteria-neuro-immune network in PD. In particular, the present study provides two major findings: (1) a concomitance of bowel inflammation, altered IEB and changes in bacterial metabolites represent early events in α-synucleinopathy, occurring before the onset of the CNS disease; (2) the activation of immune/inflammatory signaling is likely to represent the crossroads between enteric α-syn accumulation and the impairment of IEB.

As a first step, we examined the occurrence of enteric inflammatory responses in early PD mice, and we found an increase in colonic IL-1β and TNF levels as well as enteric glia activation in Tg mice since 3 months of age. Consistently with our findings, several studies have shown that PD patients at different stages of the disease were characterized by colonic inflammation^8,25^.

Of note, in a previous study, we demonstrated that A53T α-syn mice were characterized by an increase in enteric α-syn expression in myenteric neurons, as well as colonic dysmotility, occurring before the onset of typical motor symptoms and α-syn pathology in the brain, thus suggesting that the accumulation of α-syn and colonic dysmotility represent an early sign of α-syn-driven pathology without concomitant CNS involvement^9^. In this context, it is noteworthy that the accumulation of α-syn and the increase in IL-1β levels in colonic tissues from Tg mice suggest the involvement of the inflammasome signaling in the onset of enteric inflammation^26,27^. Therefore, we evaluate the activity of caspase-1, regarded as an indirect index of canonical inflammasome activation signaling^28^. Our data indicate the occurrence of a significant increase in colonic caspase-1 activity in Tg mice at 3 and 9 months of age. Interestingly, a similar pattern has been detected in brain tissues from PD patients, where α-syn deposition, nucleotide-binding oligomerization domain leucine rich repeat and pyrin domain-containing protein 3 (NLRP3)-caspase-1 signaling activation and IL-1β release were observed^27^. In addition, an increase in circulating α-syn levels in PD patients was found to correlate with an overactivation of inflammasome signaling^26^. Thus, these findings point out that the activation of canonical inflammasome signaling contributes to enteric inflammation in PD mice since the earliest stages of the disease.

Of interest, the occurrence of enteric immune/inflammatory responses could impair IEB integrity and permeability^13,29^. Therefore, in the second part of the study, we examined the colonic expression of the tight junction proteins ZO-1 and occludin, regarded as pivotally involved in the maintenance of IEB integrity^30^. The results showed significant changes in expression patterns of tight junction proteins in colonic tissues from Tg mice. A similar pattern has been detected both in patients with early PD and in animal models with PD induced by toxins, characterized by alterations of occludin and ZO-1 expression^6,31^. However, further experiments should be performed in order to better clarify the age-dependent alterations of tight junction protein expression in pre-symptomatic PD mice.

Of note, changes in tight junction protein expression could alter intestinal permeability, and accordingly, we detected an increase in circulating lipopolysaccharide-binding protein (LBP) levels in Tg animals at 3 months of age, while no changes were observed at 6 and 9 months. This pattern could result from an early increased translocation of LPS into the intestinal mucosa and systemic circulation. Subsequently, LPS can activate cell surface pattern receptors such as CD14 and TLR-4, contributing to promote innate immune/inflammatory responses, as well as be internalized by immune cells, thus determining its decrease in serum, as previously postulated^14,32,33^.

Bowel inflammation and alterations of intestinal mucosal barrier could shape gut microbiota composition and function, mainly characterized by decreased production of SCFAs, including butyrate and propionate. In particular, butyrate and propionate are regarded as a fermentation product by enteric bacteria contributing to preserve mucosal barrier integrity, modulate immune/inflammatory responses, and regulate colonic motility^13,34,35^. Therefore, in a subsequent step, we assessed the levels of butyrate and propionate in the stools from Tg mice. Our results showed a decrease in butyrate levels at 3 months, while propionate concentrations were decreased at 6 and 9 months of age. These results are in line with previous studies showing a decrease in butyrate and propionate levels in PD patients, that could further contribute to the alterations of IEB and bowel inflammation^6,36,37^.

Of interest, it is noteworthy that TLRs, including TLR-2, expressed on immune/inflammatory, intestinal epithelial as well as on enteric neurons and glial cells, are involved in the triggering immune/inflammatory responses, in the maintenance IEB integrity as well as in the shaping gut microbiota^16,17^. In this respect, in the fourth part of the study we evaluated the colonic expression of TLR-2 in Tg mice and found a decrease at 3 months, an increase at 6 months and a normalization at 9 months. Interestingly, this pattern is in line with the ZO-1 expression and butyrate levels, thus leading to hypothesize that, in PD mice, TLR-2 could contribute to impair IEB, and, in turn, shape enteric bacterial metabolites. These findings are in keeping with previous studies showing that TLR-2 on intestinal epithelial cells influences ZO-1 distribution and IEB permeability. In particular, TLR-2 activation was found to increase transepithelial resistance in Caco-2 cells through apical redistribution of ZO-1^38,39^.

In an attempt of characterizing the molecular mechanisms underlying the interplay between early α-syn accumulation, the activation of immune/inflammatory responses and IEB alterations, we performed in vitro experiments in the LPS-primed PMA-differentiated THP-1 cell line^40^, and in Caco-2 epithelial cells^41^. In particular, we tested the ability of α-syn of inducing the release of IL-1β through direct activation of canonical caspase-1 dependent inflammasome signaling^27^, as well as the influence of such cytokine in the alteration of occludin and ZO-1 expression in Caco-2 cells incubated with supernatant from stimulated THP-1 cells. Interestingly, co-treatment with LPS/α-syn promoted IL-1β release in the supernatant of THP-1 cells and the THP-1-derived supernatant (containing high levels of IL-1β) decreased significantly ZO-1 and occludin expression when applied to Caco-2 cells. Such an effect was abrogated when THP-1 cells were incubated with caspase-1 inhibitor as well as when Caco-2 cells were treated with IL-1β receptor antagonist anakinra. Of note, the direct incubation of LPS and/or α-syn did not induce IL-1β release and did not modify ZO-1 and occludin expression in Caco-2 cells. These results, show that α-syn monomers compromise IEB integrity through the activation of canonical caspase-1-dependent inflammasome signaling.

However, though our intent was to demonstrate whether early enteric α-syn accumulation (monomers) compromised IEB through the direct activation of caspase-1-dependent inflammasome signaling and the consequent IL-1β release, an integrated assessment of the effects of monomers, oligomers or fibrils of both wild-type and A53T α-syn in cell cultures remains to be clarified and could represent the logical continuation in this research topic.

Of note, the incubation of butyrate in Caco-2 cells did not prevent the reduction of ZO-1 and occludin expression induced by IL-1β-containing THP-1 supernatant. These findings are in line with previous studies, performed in intestinal epithelial cells treated with inflammatory stimuli (i.e., TNF, interferon gamma (IFN-γ) and soybean oil-based lipid emulsion), showing that butyrate did not modify the expression of TJs, such as ZO-1 and occludin, while it improved the IEB permeability^42,43^. Such an effect was ascribed to the ability of butyrate of promoting the translocation of the TJs from the cytoplasm to cell surface, with consequent increase of transepithelial electrical resistance^44–47^.

Of note, the incubation of LPS and/or α-syn did not affect TLR-2 expression in both THP-1 and Caco-2 cells, suggesting that in vivo changes in TLR-2 expression did not depend on α-syn accumulation, while they could result from the activation of other inflammatory pathways. In this respect, given the relevance of the interplay among TLR-2, immune/inflammatory response and IEB impairment^4^ as well as between TLR-2 and α-syn in PD^48^, in vivo and in vitro experiments should be implemented to elucidate the role of TLR-2 in such framework.

**Fig. 6:**
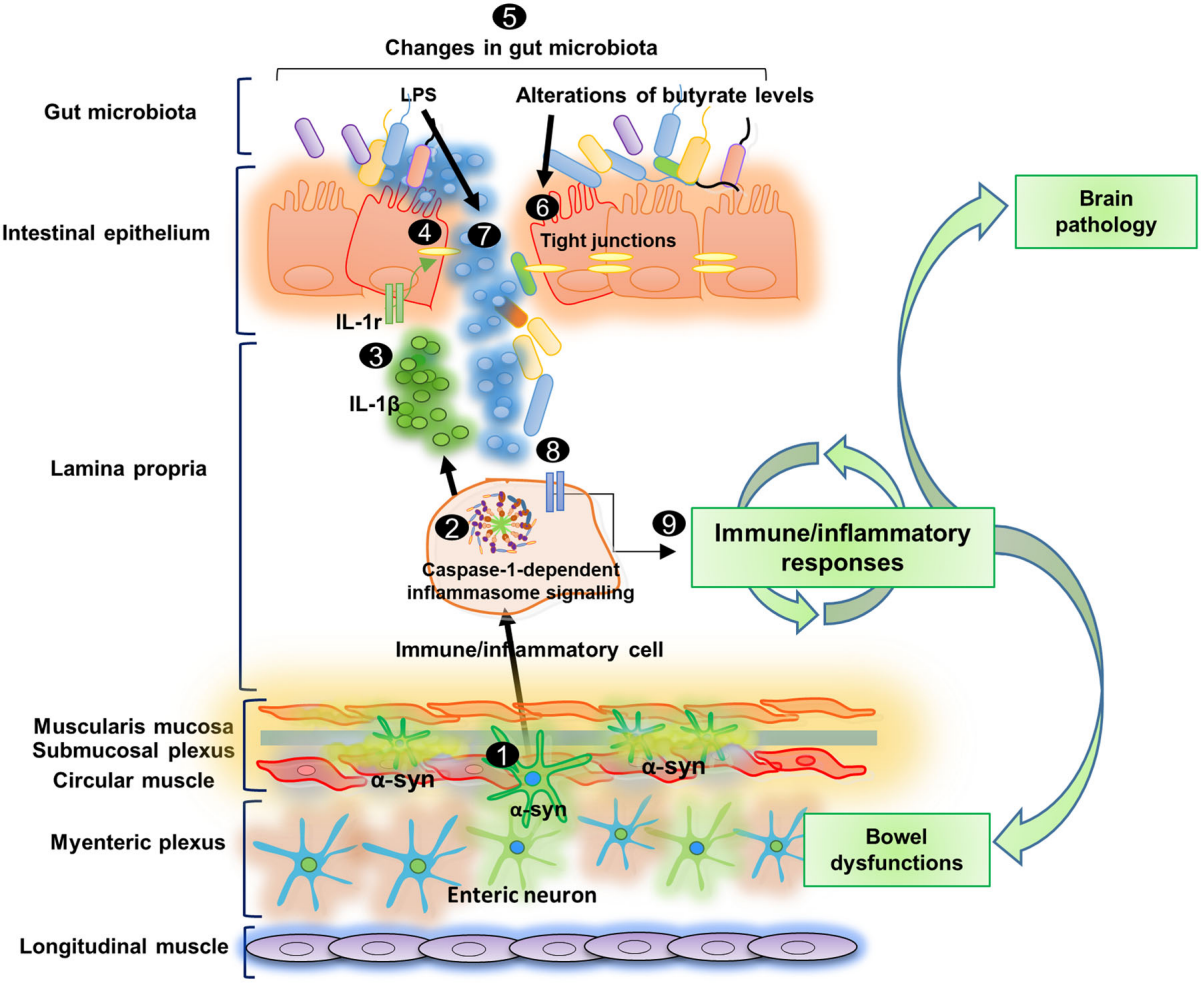
Schematic representation of pathophysiological intestinal paths in presymptomatic A53T PD mice. In the very early stages of PD before CNS pathology, the enteric α-syn accumulation **1** can promote the activation of immune/inflammatory signaling, including canonical caspase-1-dependent inflammasome pathways **2**, with consequent massive release of IL-1β **3**, which, in turn, impairs IEB **4**, through the activation of IL-1 receptors on intestinal epithelial cells. In this setting, bowel inflammation and the impaired IEB can induce changes in SCFA levels **5**, characterized by alterations of butyrate levels, that could contribute to IEB impairment **6**, and, an increase in LPS concentration, which, translocating into the intestinal mucosa **7** could further contribute to the activation of immune/inflammatory pathways **8**, thus generating a vicious circle that might lead to the chronicization of inflammatory processes and contribute both to intestinal symptoms and brain pathology **9**.

Based on these findings, it is conceivable that, in the very early stages of PD, before CNS pathology, enteric α-syn accumulation promotes the activation of immune/inflammatory signaling, including canonical inflammasome pathways, with massive release of IL-1β, and subsequent impairment of IEB integrity and changes in gut microbiota metabolite production. Afterwards, LPS translocation into the intestinal mucosa could further promote the activation of immune/inflammatory pathways, thus generating a vicious circle that might lead to the chronicization of inflammatory processes and contribute both to intestinal symptoms and brain pathology (Fig. 6). However, this is a descriptive/correlative paper and further studies in pre-symptomatic Tg mice which will undergo in vivo treatment with anti-inflammatory drugs, including inflammasome inhibitors, are required to directly test this hypothesis, and establish the role of the interplay among α-syn and bacteria-immune network in PD.

In conclusion, the present study shows that early enteric α-syn accumulation contributes to compromise IEB through the direct activation of canonical caspase-1-dependent inflammasome signaling. These changes could contribute both to bowel symptoms as well as systemic and central neuroinflammatory and neurodegenerative processes. For these reasons, these observations might represent a promising basis for the development of suitable pharmacological approaches targeting bowel IEB dysfunction and immune/inflammatory response, potentially useful for the management of intestinal dysfunctions associated with PD.

## Methods

### A53T α-syn Tg mouse model

Mice expressing the human A53T mutant of α-syn driven by the prion promoter were used in this study, as previously reported^9,24,49,50^. This model develops adult-onset progressive motor deficits, including reduced spontaneous activity with bradykinesia, mild ataxia, and dystonia, around 1-year of age followed by rapidly progressive paralysis and death within 14–21 days from the onset of the first symptoms. Once the first symptoms appear (around 12–14 months of age), that mouse is committed to develop the full phenotype (sick).

A53T mice are characterized by neuronal degeneration and accumulation of α-syn positive lesions in the CNS, more specifically in the spinal cord and midbrain regions. In the pre-symptomatic stage, when neurological function is intact, these mice show constipation and colonic dysmotility along with accumulation of α-syn inclusions in colonic neurons starting from 3 months of age. For this study, young pre-symptomatic A53T Tg mice at 3, 6, and 9 months of age were used before the appearance of any neurological symptom and compared to nTg. In addition, sick Tg mice at 12–14 months and age-matched nTg controls were also used.

All experiments with mice were performed according to the national and international laws for laboratory animal welfare and experimentation (EU directive n. 2010/63/EU and Italian DL n. 26/2014. Authorization protocol n. 213/2020-PR).

### Evaluation of tumor necrosis factor and interleukin-1β levels in colonic tissues

The evaluation of TNF and IL-1β levels in colonic tissues were performed by ELISA kits (Abcam), as described previously^40,51^. For this purpose, colonic tissue samples (20 mg), stored previously at −80 °C, were weighed, thawed, and homogenized in 0.4 ml of phosphate-buffered saline (PBS), pH 7.2 at 4 °C, and centrifuged at 10,000 *g* for 5 min. Aliquots (100 µL) of the supernatants were then used for each test. The concentrations of TNF and IL-1β were expressed as picograms per milligram of tissue.

### Evaluation of caspase-1 activity

Caspase-1 activity in colonic tissues was measured by using the colorimetric protease assay kit (BioVision Research Products, Mountain View, CA). For this purpose, colonic tissue samples (20 mg), stored previously at −80 °C, were weighed, thawed, and homogenized in 0.4 ml of PBS, pH 7.2 and centrifuged at 10,000 *g* for 5 min. Aliquots (100 μL) of the supernatants were then used for the assay.

### Measurement of lipopolysaccharide-binding protein in plasma

Plasma LBP levels were quantified in Tg and nTg mice using a commercial ELISA kit (ab213876, Abcam). Aliquots (100 μL) of plasma were used for assay. The concentrations of LBP were expressed as nanograms per milliliter.

### Tissue preparation and western blot analysis

The colon was collected from mice and flushed of fecal content with ice-cold PBS, with proteases and phosphatases inhibitors, as described previously^9,50,52^. Tissues were minced and homogenized using a Potter-Elvehjem Grinder homogenizer on ice in 20% (w/v) TNE lysis buffer (50 mM Tris-HCl pH 7.4, 100 mM NaCl, 0.1 mM EDTA, 1% NP-40, 1% SDS, 0.1% DOC) with proteases and phosphatases inhibitors. Samples were then sonicated and boiled for 5 min at 95 °C. Total lysates were run on a 4–20% Criterion^™^ TGX^™^ Precast Midi Protein Gel (Bio-Rad, Hercules, CA, USA) and then transferred onto nitrocellulose membrane at 200 mA, o/n at 4 °C, using a carbonate transfer buffer (10 mM NaHCO_3_, 3 mM Na_2_CO_3_, 20% MeOH). Transfer efficiency was controlled by Ponceau staining. Unspecific binding sites were blocked by 30 min membranes incubation with 5% non-fat dry milk (Bio-Rad) in 1X PBS containing 0.01% Tween-20 (PBS-T) at room temperature (RT). Membranes were then incubated with the specific primary antibody dissolved in 2.5% non-fat dry milk in PBS-T, o/n at 4 °C. The following primary antibodies were used: synuclein (α-syn, 1:5000; BD Biosciences, NJ, USA), ZO-1 (1:500; ab96587, Abcam), occludin (1:500; ab31721, Abcam) and TLR-2 (1:500; Santa Cruz, Dallas, TX, USA). Membranes were washed with PBS-T and incubated for 1 h at RT with the appropriate horseradish peroxidase-conjugated secondary antibody in 2.5% non-fat milk in PBS-T. The chemiluminescent signals were visualized using a CCD-based Bio-Rad Molecular Imager ChemiDoc System and resulting band intensities were analyzed using Image Lab software (Bio-Rad). All blots derive from the same experiment and they were processed in parallel.

### Samples preparation and measurement of fecal SCFAs

Feces were collected and immediately stored at −80 °C until further processing. Fecal samples were prepared by using up to 100 mg of frozen material. Feces were processed with 700 µl of 1-butanol (ACROS Organics, Fisher Scientific) followed by a gentle vortexing for 1 min and then kept at RT for 50 min. After centrifugation at 300 *g* for 10 min, the supernatant was transferred to another glass tube containing 1 ml of Boron trifluoride-1-butanol solution (~10% in 1-butanol). Tubes were vortexed and kept at RT for 30 min. 2.5 ml of hexane, 1 ml of distilled water and 0.2 ml of saturated sodium chloride solution were then added to the preparations and samples were vortexed and kept at RT for 50 min. An aliquot of the supernatant, hexane containing the butylated SCFA, was diluted with methanol (1:4).

The identification and quantification were carried out by liquid chromatography-tandem mass spectrometry (LC-MS/MS). An Agilent 1100 HPLC system (Agilent, Palo Alto, CA, USA) was coupled to a mass spectrometry Agilent Technologies QQQ triple quadrupole 6420 equipped with a ESI source, using positive mode (ESI + ). A C-18 Zorbax Eclipse Plus column (Agilent, Palo Alto, CA, USA) with 5 μm particle size and 50 × 4.6 mm was used with a mobile phase of CH_3_OH/H_2_O/CHOOH (70/30/0.1, v/v/v) at a flow rate of 0.5 ml/min. N_2_ was used as a nebulizing gas with a pressure of 50 psig, drying gas temperatures 300 °C and flow of 11 L/min, and 4000 V capillary voltage. For each standard, the precursor ion [M + H]^+^ was determined during a full scan in MS and subsequently the obtained product ion (PI) was monitored for each transition in MRM mode in MS/MS. Parameters of source, such as cone voltage or fragmentor (CV) and collision energy (CE) have been optimized for each MRM transition. Butyl propionate and butyl butyrate transitions were [131→ 75] and [145 →89] respectively.

Data were acquired by the MassHunter workstation acquisition software and analyzed with MassHunter software for qualitative and quantitative analysis. Butyric and propionic acid (Supelco) and butyl butyrate and butyl propionate, Boron trifluoride-1-butanol solution (~10% in 1-butanol), formic acid, n-hexane, water and methanol, LiChrosolv gradient grade for liquid chromatography were purchased from Sigma Chemicals Co. (St. Louis, MO, USA).

### Immunofluorescence on colonic tissues

Frozen sections of the distal colon were embedded in Tissue-Tek^®^ OCT (Sakura, The Netherlands), cut at the cryostat in serial 12 μm sections and mounted on Super-Frost Plus glass slide (Thermo fisher). For immunostaining slides were incubated with blocking solution [3.5% fat dry milk, 0.3% Triton X-100 (Tx-100), 6% normal goat serum (NGS) in PBS] for 1 h at RT and then incubated with TLR-2 (Santa Cruz, Dallas, TX, USA) and GFAP (ab7260, Abcam) antibody o/n at RT in blocking buffer. Slides were washed twice in PBS and incubated with Alexa Fluor secondary antibodies (Thermo Fisher) in PBS containing 1.5% NGS, 0.3% Tx-100 for 1 h at RT. Sections were counterstained with Dapi and mounted on a glass slide using Fluormount (Sigma-Aldrich). Image acquisition was carried out using a Zeiss Apotome fluorescent microscope, using a 20× objective.

### Immunofluorescence on brain tissues

For immunofluorescence analysis, mice were transcardially perfused with 4% paraformaldehyde in PBS (pH 7.4). Perfused brains were dissected, post-fixed, and cryoprotected in 30% sucrose in PBS. The brains were sectioned to obtain 30 µm-thick coronal sections that were stored in maintenance solution (30% sucrose, 0.1% NaN_2_ in PBS) at 4 °C until used. Selected brain sections from the midbrain region were blocked for 30 min at RT in 10% NGS, 0.3% Triton X-100 in PBS, followed by an o/n incubation at 4 °C with 1:500 rabbit anti-IBA1 (019-19741, Wako, Richmond VA, USA) in 5% NGS, 0.3% Triton X-100 in PBS. Sections were then incubated with 1:500 anti-rabbit secondary antibody conjugated to Alexa Fluor-488 fluorophore (A11008, Thermo Fisher Scientific, MA, USA) diluted in 5% NGS in PBS for 3 h at RT. Images were acquired using a 40× objective of Leica TCS SP confocal laser-scanning microscope (Leica, Germany). Images elaboration and cell density analysis were obtained using ImageJ (NIH).

### Cell cultures

Human intestinal epithelial Caco-2 cell line were routinely cultured in Dulbecco’s Modified Eagle Medium supplemented with 20% fetal bovine serum (FBS), 2 mM glutamine and 100 unit/ml penicillin-streptomycin. Human monocytic THP-1 cell line was grown in RPMI-1640 supplemented with 10% FBS and 100 unit/ml penicillin-streptomycin at 37 °C in a humidified atmosphere of 5% CO_2_. THP-1 were kindly donated by Prof Veit Hornung (Ludwig Maximilian, University of Munich) and Caco-2 cells were kindly donated by Prof Guido Bocci (Department of clinical and Experimental Medicine, University of Pisa).

### Cell treatments

THP-1 cells were seeded at density of 1 × 10^7^ cells in 100 mm Petri dish and treated with PMA (0.5 µM). After 3 h, the medium was removed, fresh media was added, and cells were incubated overnight (37 °C, 5% CO_2_).

In the first series of experiments, cells were treated with monomeric wild type (WT) α-syn (1 µM, 24 h) (S7820, Sigma Aldrich) or LPS (1 µg/ml, 4 h) to induce pro-IL-1β expression^53–56^.

In the second series of experiments, cells were LPS-primed (1 µg/ml, 4 h) before treatment with Nig (a standard NLRP3 inflammasome activator, 10 µM, 1 h) or α-syn (1 µM, 24 h)^57^.

Of note, LPS treatment promotes pro-IL-1β and NLRP3 transcription through nuclear factor-κB activation (first step of inflammasome activation)^58^ while α-syn, including monomers, oligomers and fibrils, is regarded to induce inflammasome activation by initiating assembly of a multiprotein complex consisting of NLRP3, the adaptor protein ASC, and pro-caspase-1, that, in turn, lead to cleavage of caspase-1 in its active form and the consequent IL-1β release. Therefore, the co-treatment with LPS and α-syn promotes the first and the second step underlying canonical inflammasome activation, contributing to the IL-1β processing and release^27,59^.

In the third series of experiments, LPS-primed (1 µg/ml, 4 h) cells were treated for 1 h with caspase-1 inhibitor (YVAD, 50 µM) before the addition of nigericin (10 µM, 1 h) or α-syn (1 µM, 24 h). Controls were run in parallel.

After THP-1 stimulation, the medium was collected and centrifuged for 5 min at 800 *rpm* to obtain cell-free supernatants. Cell supernatants (conditioned medium) were used to treat Caco-2 cells, previously seeded at density of 5 × 10^5^ cells in six-well plate. After 72 h, Caco-2 cells were lysed.

For the experiments with IL-1β receptor antagonist, Caco-2 cells were treated for 72 h with 100 ng/ml anakinra (Amgen, Thousand Oaks, CA, USA) in the presence of α-syn plus LPS conditioned medium.

In addition, to characterize the effect of butyrate on tight junction expression, Caco-2 cells were treated with conditioned medium derived from THP-1 cells treated with α-syn plus LPS, in the absence or in the presence of 2 mM butyrate (sodium butyrate, 567430 Sigma Aldrich) for 72 h. Experimental protocol and the concentration of butyrate were selected in accordance with previous studies^45,46,60^. Controls were run in parallel.

### Assessment of IL-1β release in THP-1 cells

The release of IL-1β was measured by ELISA kit (R&D system), as previously described^40,61^. After cell stimulation, the medium was collected and centrifuged for 5 min at 800 *rpm* to obtain cell-free supernatants. Aliquots of 100 μl were used for the test. IL-1β concentration was expressed as picograms per milliliter.

### Western Blot in cell lysates

Cells were lysed as previously described^62,63^. Proteins were quantified with the Bradford assay. Proteins were separated onto a pre-cast 4–20% polyacrylamide gel (Mini-PROTEAN^®^ TGX gel, Bio-rad) and transferred to PVDF membranes (Trans-Blot^®^ TurboTM PVDF Transfer packs, Bio-rad). Membranes were blocked with 3% BSA diluted in Tris-buffered saline (TBS, 20 mM Tris-HCl, PH 7.5, 150 mM NaCl) with 0.1% Tween 20. Primary antibodies against β-actin (1:5000; ab8227, Abcam), ZO-1 (1:300; ab96587, Abcam), occludin (1:5000; ab31721, Abcam) and TLR-2 (1:500; Santa Cruz, Dallas, TX, USA) were used. Secondary antibodies were obtained from Abcam (1:5000; anti-mouse ab97040 and anti-rabbit ab6721). Protein bands were detected with ECL reagents (Clarity^TM^ Western ECL Blotting Substrate, Bio-rad). Densitometry was performed by ImageJ software. All blots derive from the same experiment and they were processed in parallel.

### Statistical analysis

The results are presented as mean ± S.E.M. of at least three independent experiments. Outliers were defined as values that exceed the distance from the median value by 50%. The analysis of the Gaussian distribution was carried out using Shapiro–Wilk normality test. The significance of differences was evaluated by one-way analysis of variance (ANOVA) followed by Tukey post hoc test or Fisher, or two-way analysis of variance (ANOVA) followed by Tukey’s post hoc test, were appropriate. *P* values < 0.05 were considered significantly different. All statistical procedures were performed by commercial software (GraphPad Prism, version 7.0 from GraphPad Software Inc., San Diego, CA, USA).

### Reporting summary

Further information on research design is available in the Nature Research Reporting Summary linked to this article.

## Supplementary information

Supplementary Figures

REPORTING SUMMARY

## Data Availability

The data that supports the findings of this study are available from the corresponding authors upon reasonable request.

## References

[1] Fasano A, Visanji NP, Liu LW, Lang AE, Pfeiffer RF (2015). Gastrointestinal dysfunction in Parkinson’s disease. Lancet Neurol..

[2] Pellegrini C (2016). Intestinal dysfunction in Parkinson’s disease: lessons learned from translational studies and experimental models. Neurogastroenterol. Motil.: Off. J. Eur. Gastrointest. Motil. Soc..

[3] Pellegrini C (2015). Gastric motor dysfunctions in Parkinson’s disease: current pre-clinical evidence. Parkinsonism Relat. Disord..

[4] Pellegrini C, Antonioli L, Colucci R, Blandizzi C, Fornai M (2018). Interplay among gut microbiota, intestinal mucosal barrier and enteric neuro-immune system: a common path to neurodegenerative diseases?. Acta Neuropathologica.

[5] Keshavarzian A (2015). Colonic bacterial composition in Parkinson’s disease. Mov. Disord.: Off. J. Mov. Disord. Soc..

[6] Perez-Pardo P (2019). Role of TLR4 in the gut-brain axis in Parkinson’s disease: a translational study from men to mice. Gut.

[7] Yang X, Qian Y, Xu S, Song Y, Xiao Q (2017). Longitudinal Analysis of Fecal Microbiome and Pathologic Processes in a Rotenone Induced Mice Model of Parkinson’s Disease. Front. Aging Neurosci..

[8] Devos D (2013). Colonic inflammation in Parkinson’s disease. Neurobiol. Dis..

[9] Rota L (2019). Constipation, deficit in colon contractions and alpha-synuclein inclusions within the colon precede motor abnormalities and neurodegeneration in the central nervous system in a mouse model of alpha-synucleinopathy. Transl. Neurodegeneration.

[10] Strowig T, Henao-Mejia J, Elinav E, Flavell R (2012). Inflammasomes in health and disease. Nature.

[11] Lopez-Castejon G (2013). Deubiquitinases regulate the activity of caspase-1 and interleukin-1beta secretion via assembly of the inflammasome. J. Biol. Chem..

[12] Gu XL (2010). Astrocytic expression of Parkinson’s disease-related A53T alpha-synuclein causes neurodegeneration in mice. Mol. Brain.

[13] Pellegrini C (2020). Microbiota-gut-brain axis in health and disease: Is NLRP3 inflammasome at the crossroads of microbiota-gut-brain communications?. Prog. Neurobiol..

[14] Gutsmann T (2001). Dual role of lipopolysaccharide (LPS)-binding protein in neutralization of LPS and enhancement of LPS-induced activation of mononuclear cells. Infect. Immun..

[15] D’Antongiovanni V (2020). Intestinal epithelial barrier and neuromuscular compartment in health and disease. World J. Gastroenterol..

[16] Maynard CL, Elson CO, Hatton RD, Weaver CT (2012). Reciprocal interactions of the intestinal microbiota and immune system. Nature.

[17] Brun P (2013). Toll-like receptor 2 regulates intestinal inflammation by controlling integrity of the enteric nervous system. Gastroenterology.

[18] Chanput W, Mes JJ, Wichers HJ (2014). THP-1 cell line: an in vitro cell model for immune modulation approach. Int. Immunopharmacol..

[19] Lopez-Castejon G, Brough D (2011). Understanding the mechanism of IL-1beta secretion. Cytokine Growth Factor Rev..

[20] Martin-Sanchez F (2016). Inflammasome-dependent IL-1beta release depends upon membrane permeabilisation. Cell Death Differ..

[21] White AJ (2018). The Peripheral Inflammatory Response to Alpha-Synuclein and Endotoxin in Parkinson’s Disease. Front. Neurol..

[22] Al-Sadi R, Ye D, Said HM, Ma TY (2010). IL-1beta-induced increase in intestinal epithelial tight junction permeability is mediated by MEKK-1 activation of canonical NF-kappaB pathway. Am. J. Pathol..

[23] Al-Sadi R (2013). Mechanism of IL-1beta modulation of intestinal epithelial barrier involves p38 kinase and activating transcription factor-2 activation. J. Immunol..

[24] Lee MK (2002). Human alpha-synuclein-harboring familial Parkinson’s disease-linked Ala-53 -> Thr mutation causes neurodegenerative disease with alpha-synuclein aggregation in transgenic mice. Proc. Natl Acad. Sci. USA.

[25] Qin XY, Zhang SP, Cao C, Loh YP, Cheng Y (2016). Aberrations in Peripheral Inflammatory Cytokine Levels in Parkinson Disease: A Systematic Review and Meta-analysis. JAMA Neurol..

[26] Chatterjee K (2020). Inflammasome and alpha-synuclein in Parkinson’s disease: a cross-sectional study. J. Neuroimmunol..

[27] Gordon, R. et al. Inflammasome inhibition prevents alpha-synuclein pathology and dopaminergic neurodegeneration in mice. *Sci. Transl. Med.* **10**, 10.1126/scitranslmed.aah4066 (2018). 10.1126/scitranslmed.aah4066 PMC6483075 30381407

[28] McKenzie BA (2018). Caspase-1 inhibition prevents glial inflammasome activation and pyroptosis in models of multiple sclerosis. Proc. Natl Acad. Sci. USA.

[29] Benvenuti, L. et al. Enteric Glia at the Crossroads between Intestinal Immune System and Epithelial Barrier: Implications for Parkinson Disease. *Int. J. Mol. Sci.* **21**, 10.3390/ijms21239199 (2020). 10.3390/ijms21239199 PMC7730281 33276665

[30] Anderson JM, Van Itallie CM (2009). Physiology and function of the tight junction. Cold Spring Harb. Perspect. Biol..

[31] Clairembault T (2015). Structural alterations of the intestinal epithelial barrier in Parkinson’s disease. Acta Neuropathologica Commun..

[32] Wurfel MM, Kunitake ST, Lichenstein H, Kane JP, Wright SD (1994). Lipopolysaccharide (LPS)-binding protein is carried on lipoproteins and acts as a cofactor in the neutralization of LPS. J. Exp. Med..

[33] Gegner JA, Ulevitch RJ, Tobias PS (1995). Lipopolysaccharide (LPS) signal transduction and clearance. Dual roles for LPS binding protein and membrane CD14. J. Biol. Chem..

[34] Soret R (2010). Short-chain fatty acids regulate the enteric neurons and control gastrointestinal motility in rats. Gastroenterology.

[35] Parada Venegas D (2019). Short Chain Fatty Acids (SCFAs)-Mediated Gut Epithelial and Immune Regulation and Its Relevance for Inflammatory Bowel Diseases. Front. Immunol..

[36] Scheperjans F (2015). Gut microbiota are related to Parkinson’s disease and clinical phenotype. Mov. Disord.: Off. J. Mov. Disord. Soc..

[37] Unger MM (2016). Short chain fatty acids and gut microbiota differ between patients with Parkinson’s disease and age-matched controls. Parkinsonism Relat. Disord..

[38] Cario E, Gerken G, Podolsky DK (2004). Toll-like receptor 2 enhances ZO-1-associated intestinal epithelial barrier integrity via protein kinase C. Gastroenterology.

[39] Cario E, Gerken G, Podolsky DK (2007). Toll-like receptor 2 controls mucosal inflammation by regulating epithelial barrier function. Gastroenterology.

[40] Pellegrini, C. et al. Prodromal Intestinal Events in Alzheimer’s Disease (AD): Colonic Dysmotility and Inflammation Are Associated with Enteric AD-Related Protein Deposition. *Int. J. Mol. Sci.* **21**, 10.3390/ijms21103523 (2020). 10.3390/ijms21103523 PMC7278916 32429301

[41] Al-Sadi RM, Ma TY (2007). IL-1beta causes an increase in intestinal epithelial tight junction permeability. J. Immunol..

[42] Yan JK, Gong ZZ, Zhang T, Cai W (2017). Sodium butyrate attenuates soybean oil-based lipid emulsion-induced increase in intestinal permeability of lipopolysaccharide by modulation of P-glycoprotein in Caco-2 cells. Biochem. Biophys. Res. Commun..

[43] Huang, X., Oshima, T., Tomita, T., Fukui, H. & Miwa, H. Butyrate Alleviates Cytokine-Induced Barrier Dysfunction by Modifying Claudin-2 Levels. *Biology* **10**, 10.3390/biology10030205 (2021). 10.3390/biology10030205 PMC8000923 33803334

[44] Jones SA, Butler RN, Sanderson IR, Wilson JW (2004). The effect of specific caspase inhibitors on TNF-alpha and butyrate-induced apoptosis of intestinal epithelial cells. Exp. Cell Res..

[45] Peng L, He Z, Chen W, Holzman IR, Lin J (2007). Effects of butyrate on intestinal barrier function in a Caco-2 cell monolayer model of intestinal barrier. Pediatr. Res..

[46] Peng L, Li ZR, Green RS, Holzman IR, Lin J (2009). Butyrate enhances the intestinal barrier by facilitating tight junction assembly via activation of AMP-activated protein kinase in Caco-2 cell monolayers. J. Nutr..

[47] Vancamelbeke M (2019). Butyrate Does Not Protect Against Inflammation-induced Loss of Epithelial Barrier Function and Cytokine Production in Primary Cell Monolayers From Patients With Ulcerative Colitis. J. Crohn’s Colitis.

[48] Kim C (2013). Neuron-released oligomeric alpha-synuclein is an endogenous agonist of TLR2 for paracrine activation of microglia. Nat. Commun..

[49] Martin LJ (2006). Parkinson’s disease alpha-synuclein transgenic mice develop neuronal mitochondrial degeneration and cell death. J. Neurosci.: Off. J. Soc. Neurosci..

[50] Colla E (2012). Endoplasmic reticulum stress is important for the manifestations of alpha-synucleinopathy in vivo. J. Neurosci.: Off. J. Soc. Neurosci..

[51] Pellegrini C (2018). A Comparative Study on the Efficacy of NLRP3 Inflammasome Signaling Inhibitors in a Pre-clinical Model of Bowel Inflammation. Front. Pharmacol..

[52] D’Antongiovanni, V. et al. Glial A2B Adenosine Receptors Modulate Abnormal Tachykininergic Responses and Prevent Enteric Inflammation Associated with High Fat Diet-Induced Obesity. *Cells* **9**, 10.3390/cells9051245 (2020). 10.3390/cells9051245 PMC7290602 32443525

[53] Mariathasan S (2006). Cryopyrin activates the inflammasome in response to toxins and ATP. Nature.

[54] Halle A (2008). The NALP3 inflammasome is involved in the innate immune response to amyloid-beta. Nat. Immunol..

[55] Martinon F, Petrilli V, Mayor A, Tardivel A, Tschopp J (2006). Gout-associated uric acid crystals activate the NALP3 inflammasome. Nature.

[56] Dostert C (2008). Innate immune activation through Nalp3 inflammasome sensing of asbestos and silica. Science.

[57] Pellegrini, C. et al. NLRP3 at the crossroads between immune/inflammatory responses and enteric neuroplastic remodeling in a mouse model of diet-induced obesity. *Br. J. Pharmacol.*10.1111/bph.15532 (2021). 10.1111/bph.15532 34000757

[58] Pellegrini C, Antonioli L, Lopez-Castejon G, Blandizzi C, Fornai M (2017). Canonical and Non-Canonical Activation of NLRP3 Inflammasome at the Crossroad between Immune Tolerance and Intestinal Inflammation. Front. Immunol..

[59] Pellegrini, C. et al. NLRP3 inflammasome in cardiovascular diseases: Pathophysiological and pharmacological implications. *Med. Res. Rev.*10.1002/med.21781 (2021). 10.1002/med.21781 33460162

[60] Chen T (2017). Dietary fibre-based SCFA mixtures promote both protection and repair of intestinal epithelial barrier function in a Caco-2 cell model. Food Funct..

[61] Antonioli L (2020). Colonic dysmotility associated with high-fat diet-induced obesity: Role of enteric glia. FASEB J.: Off. Publ. Federation Am. Societies Exp. Biol..

[62] Smoktunowicz N (2016). TGFbeta upregulates PAR-1 expression and signalling responses in A549 lung adenocarcinoma cells. Oncotarget.

[63] Fazzini A (2014). Altered protease-activated receptor-1 expression and signaling in a malignant pleural mesothelioma cell line, NCI-H28, with homozygous deletion of the beta-catenin gene. PloS ONE.

