# Author Correction: Enteric α-synuclein impairs intestinal epithelial barrier through caspase-1-inflammasome signaling in Parkinson’s disease before brain pathology

**DOI:** 10.1038/s41531-023-00536-7

**Published:** 2023-06-02

**Authors:** C. Pellegrini, V. D’Antongiovanni, F. Miraglia, L. Rota, L. Benvenuti, C. Di Salvo, G. Testa, S. Capsoni, G. Carta, L. Antonioli, A. Cattaneo, C. Blandizzi, E. Colla, M. Fornai

**Affiliations:** 1 Unit of Histology and Medical Embryology, Department of Clinical and Experimental Medicine, University of Pisa, Pisa, Italy; 2 Unit of Pharmacology and Pharmacovigilance, Department of Clinical and Experimental Medicine, University of Pisa, Pisa, Italy; 3 Bio@SNS Laboratory, Scuola Normale Superiore, Pisa, Italy; 4 Department of Biomedical Science, University of Cagliari, Cagliari, Italy

**Keywords:** Cellular neuroscience, Parkinson's disease

Correction to: *npj Parkinson’s Disease* 10.1038/s41531-021-00263-x, published online 12 January 2022

“In this article the wrong figure appeared as Fig. 5b. where an incorrect representative western blot was displayed for Anti-occludin 59 KDa and related-actin 49 KDa, and in Supplementary Figure 8B where an incorrect representative uncropped western blot was displayed for Anti-occludin 59 KDa.

The Figures should have appeared as shown below. The original article has been corrected.”
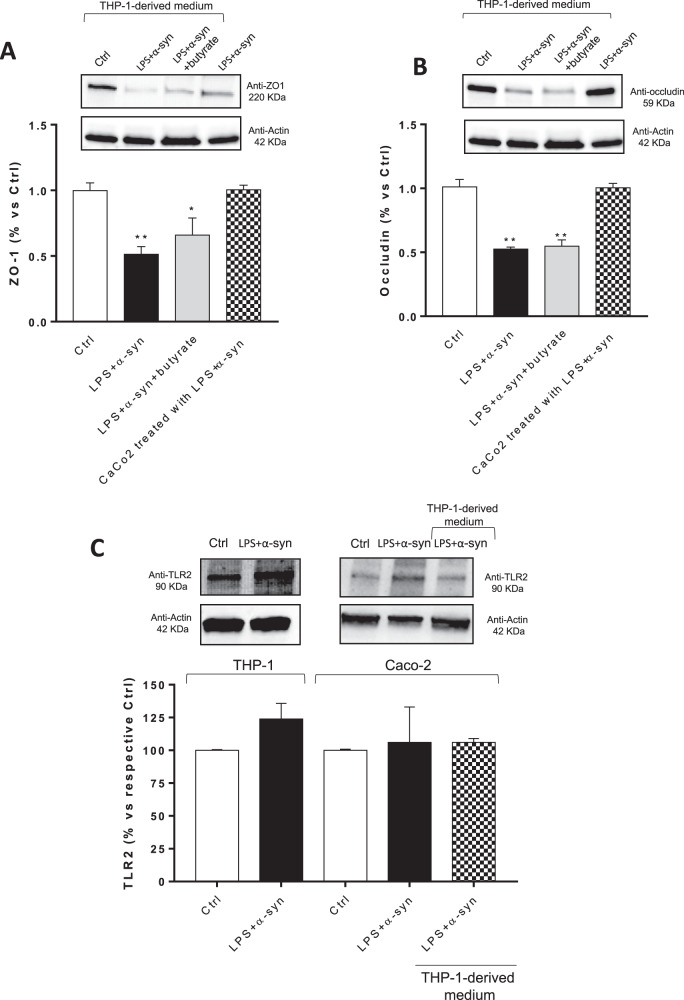

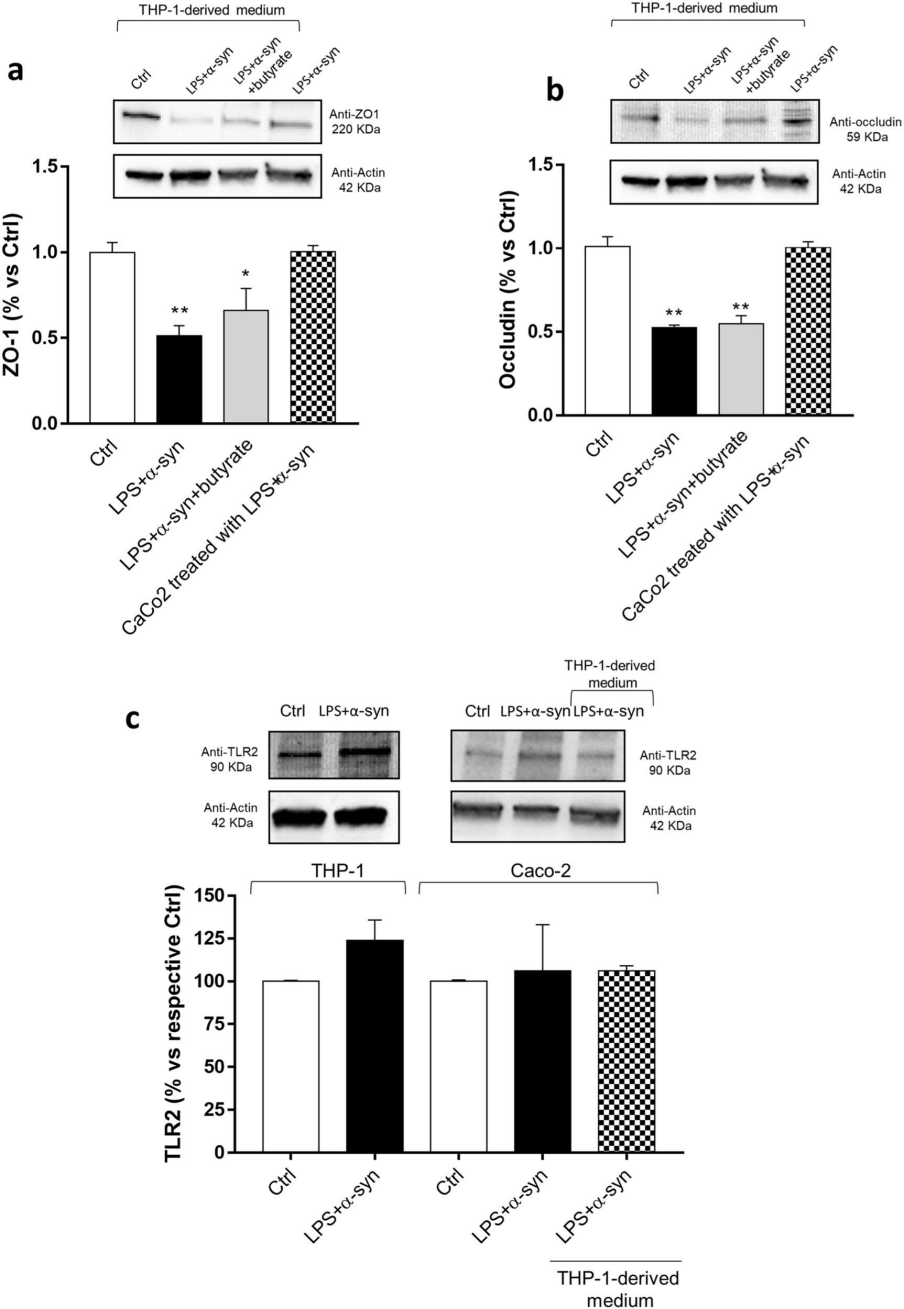


## Supplementary information

Supplementary Figure correct

