# Supplementary material for: Enteric α-synuclein impairs intestinal epithelial barrier through caspase-1-inflammasome signaling in Parkinson’s disease before brain pathology
Source: NPJ Parkinsons Dis. 2022 Jan 12;8:9. doi: 10.1038/s41531-021-00263-x (PMC8755783; doi:10.1038/s41531-021-00263-x)
Supplement: Supplementary file 1 — Supplementary Figures [file 41531_2021_263_MOESM1_ESM.pdf]

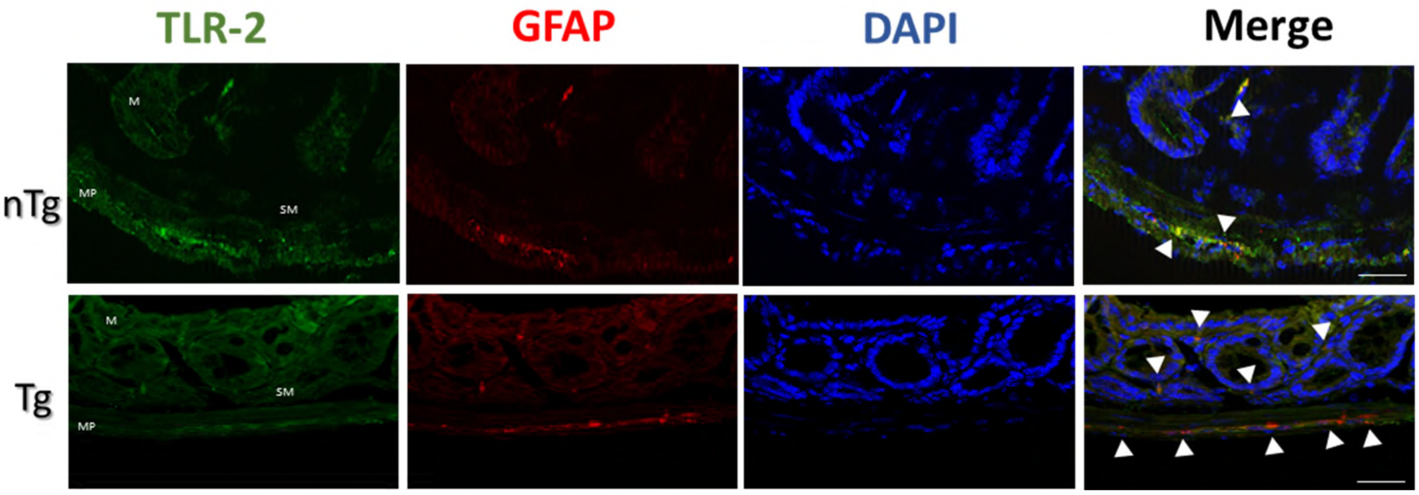

Supplementary Fig. 1 Enteric glial cells co-localize with TLR-2 in colonic tissue. Immunofluorescence staining of GFAP (enteric glial marker) and TLR-2 in colonic tissues from Tg and nTg animals at 3 months. Images were acquired with a Zeiss Apotome fluorescent microscope, with an objective 20x, scale bar =100 µm.
Abbreviations: GFAP, glial fibrillary acidic protein; nTg, non-transgenic; *Tg*, transgenic; TLR, toll like receptor.

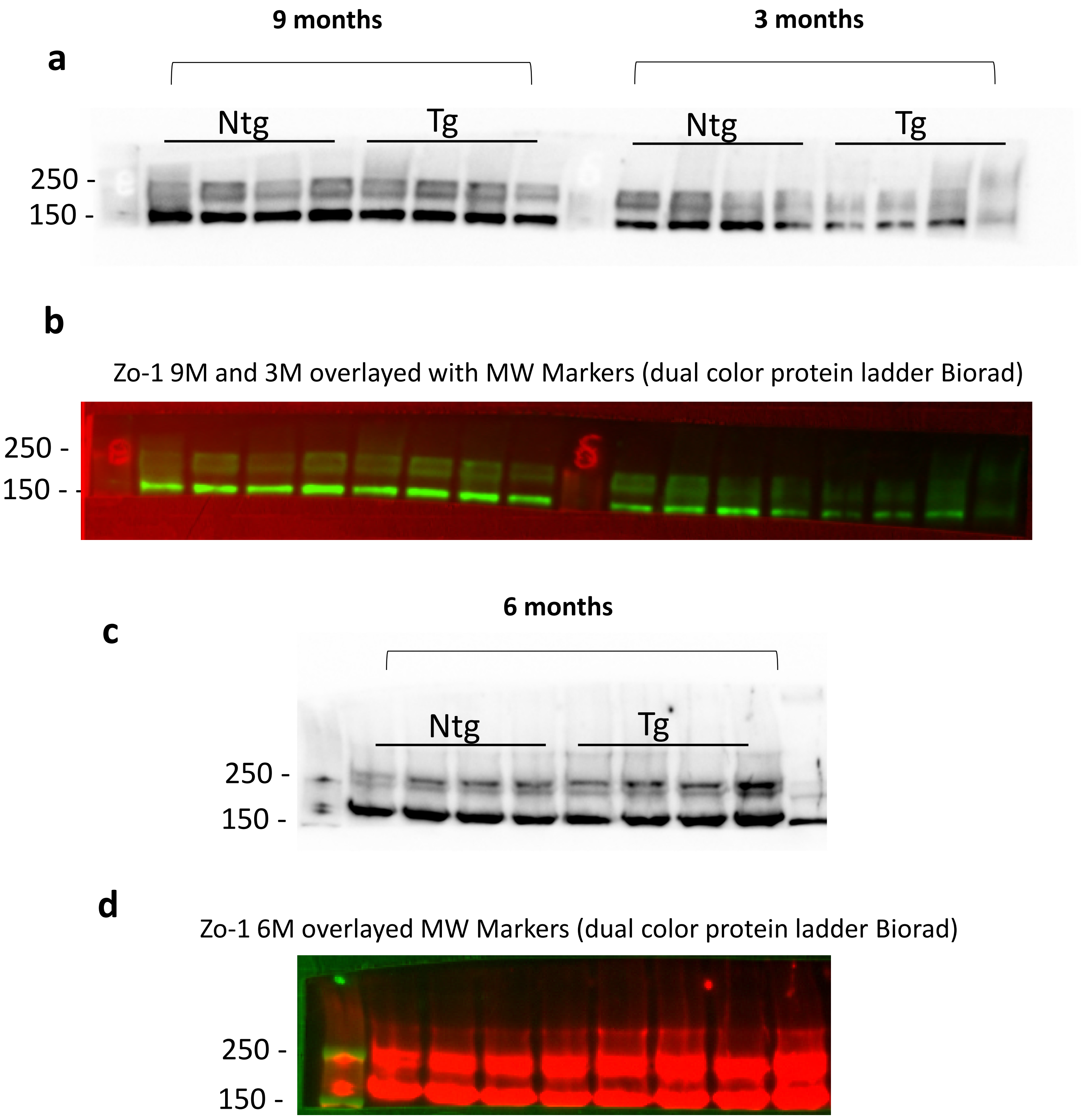

Supplementary Fig. 2 Full length Western blots shown in Fig. 2. Representative “uncropped” blots of zonulin-1 (ZO-1) expression assessed by Western blot assay in colonic tissues from nTg and Tg mice at 3 (**a, b**) , 6 (**c, d**) and 9 (**a, b**) months of age. **b, d** Western blot overlap with MW markers.

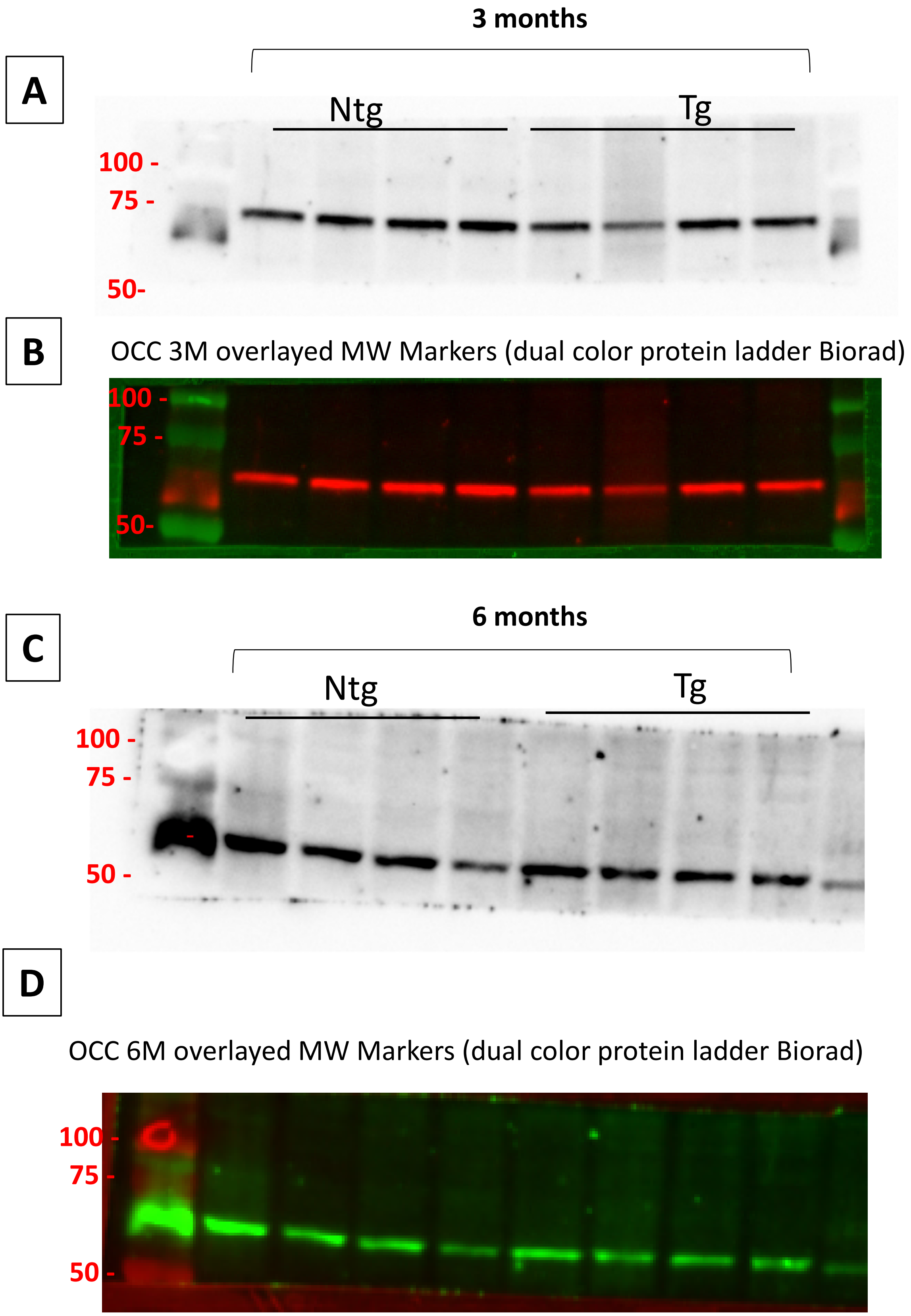

Representative "uncropped" blots of occludin (OCC) expression assessed by Western blot assay in colonic tissues from nTg and Tg mice at 3 (**A, B**) and 6 (**C, D**) months of age. (**B, D**) western blot overlap with MW markers.

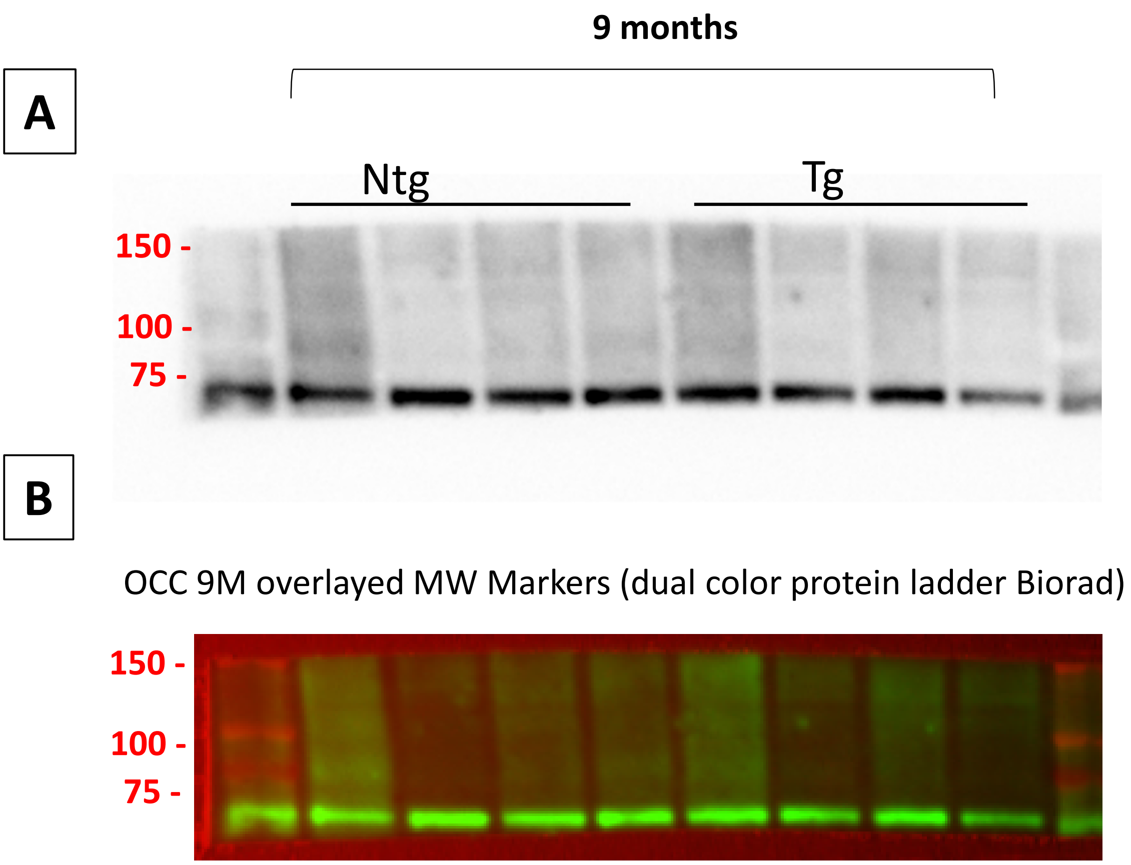

Representative “uncropped” blots of occludin (OCC) expression assessed by Western blot assay in colonic tissues from nTg and Tg mice at 9 months of age (**A, B**). (**B**) western blot overlap with MW markers.

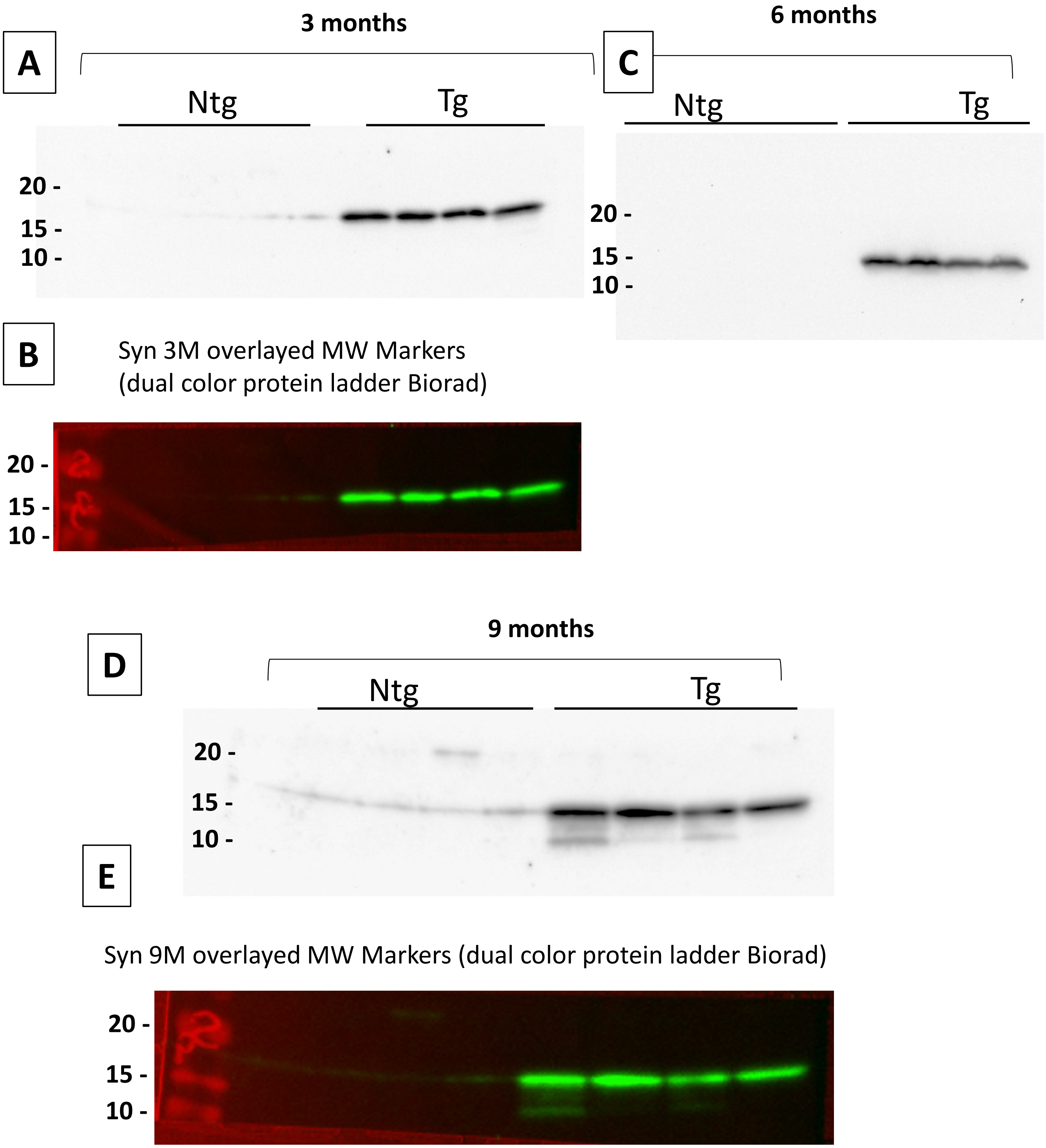

Representative “uncropped” blots of α-synuclein (α-syn) expression assessed by Western blot assay in colonic tissues from nTg and Tg mice at 3 (**A, B**), 6 (**C**) and 9 (**D, E**) months of age. (**B, E**) western blot overlap with MW markers.

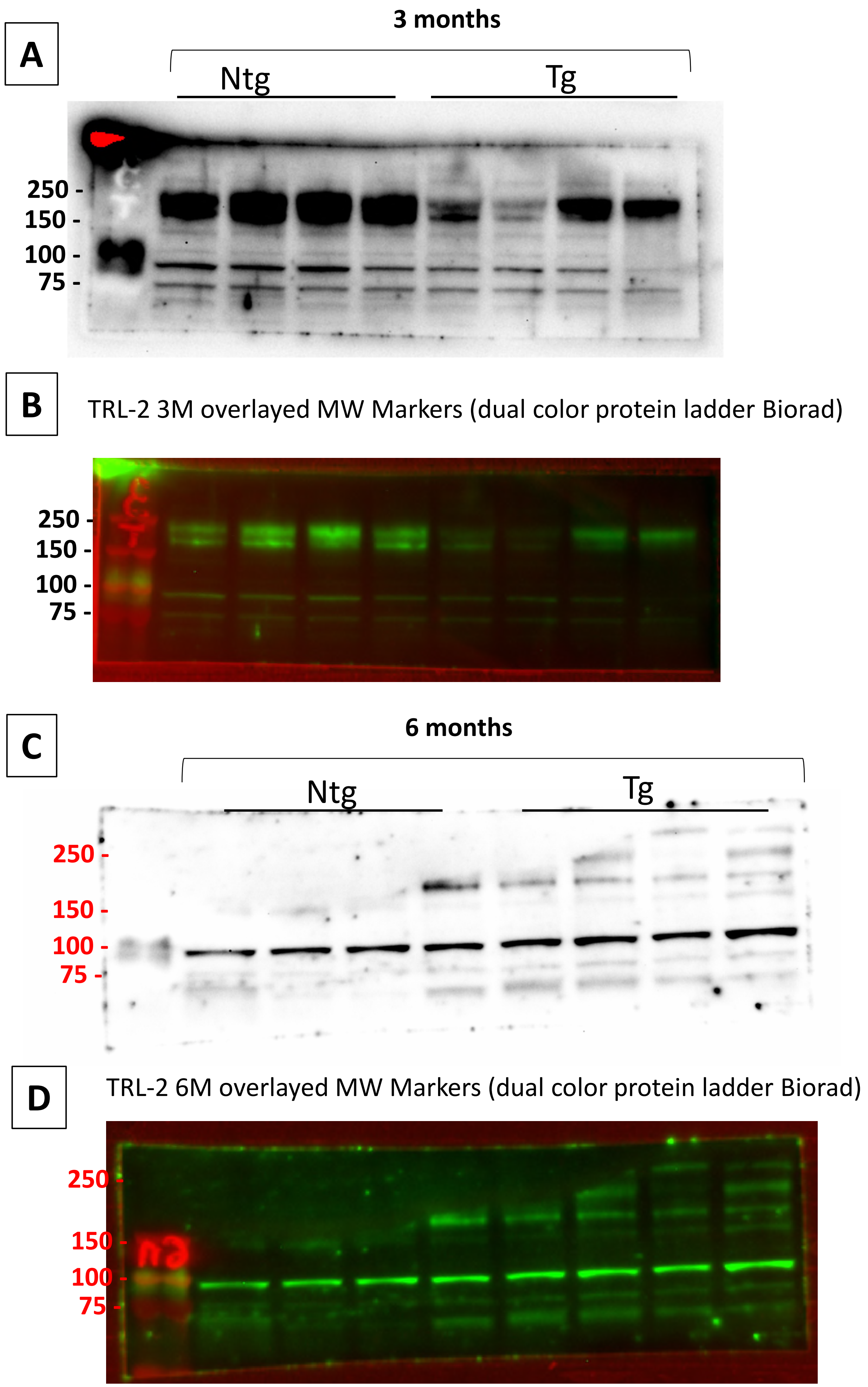

Representative “uncropped” blots of toll like receptor-2 (TLR-2) expression assessed by Western blot assay in colonic tissues from nTg and Tg mice at 3 (**A, B**) and 6 (**C, D**) months of age. (**B, D**) western blot overlap with MW markers.

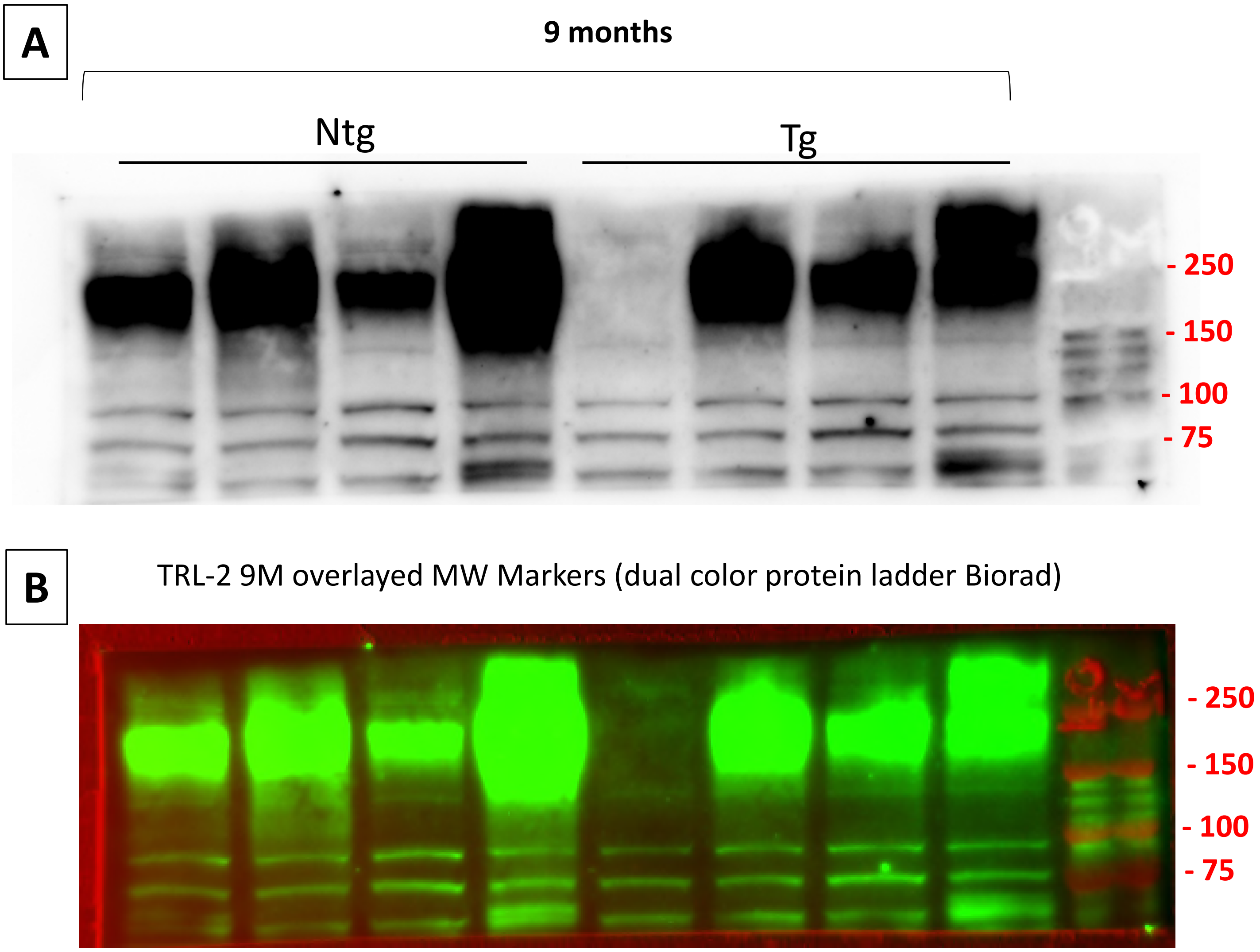

Representative “uncropped” blots of toll like receptor-2 (TLR-2) expression assessed by Western blot assay in colonic tissues from nTg and Tg mice at 9 (**A, B**) months of age. (**B**) western blot overlap with MW markers.

Composite image for marker view

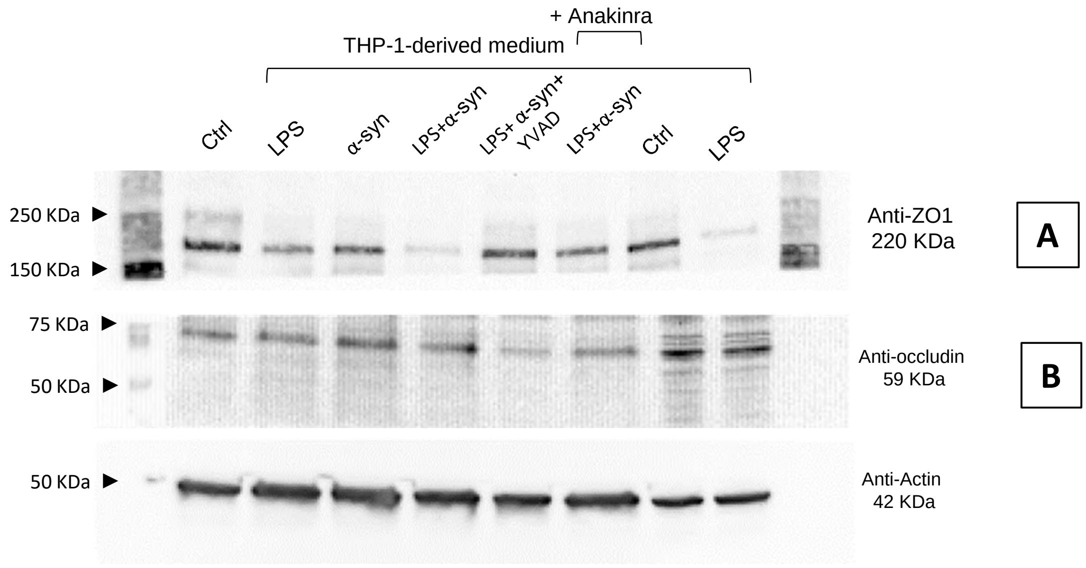

Representative “uncropped” blots of ZO-1 (**A**) and occludin (**B**) expression assessed by Western blot assay in cultured Caco-2 cells treated with conditioned medium derived from THP-1 cells. The membrane was cut for the detection of ZO-1, occludin and actin. These blots correspond to Figure 4B in the manuscript.

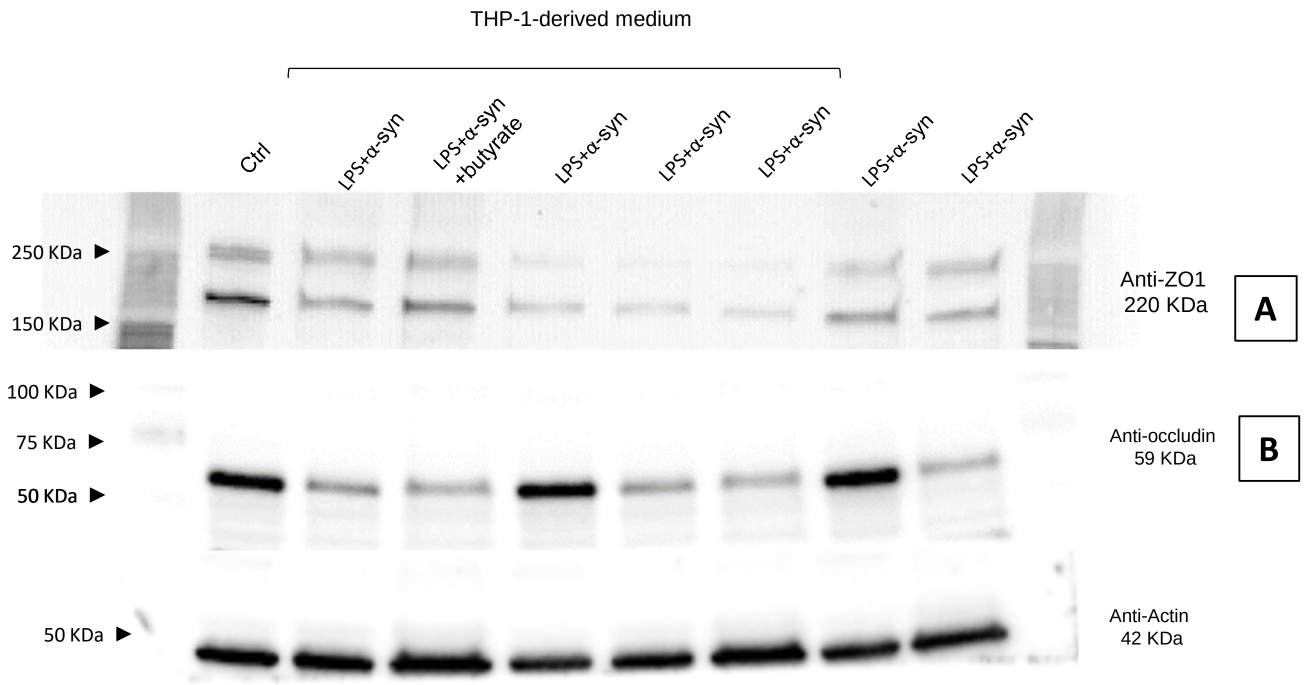

Representative “uncropped” blots of ZO-1 (**A**) and occludin (**B**) expression assessed by Western blot assay in cultured Caco-2 cells treated with conditioned medium derived from THP-1 cells treated with α-syn plus LPS, in the absence or in the presence of butyrate, or with α-syn plus LPS directly in the medium. The membrane was cut for the detection of ZO-1, occludin and actin. These blots correspond to Figure 5A e 5B in the manuscript

Composite image for marker view

THP-1 cell

A

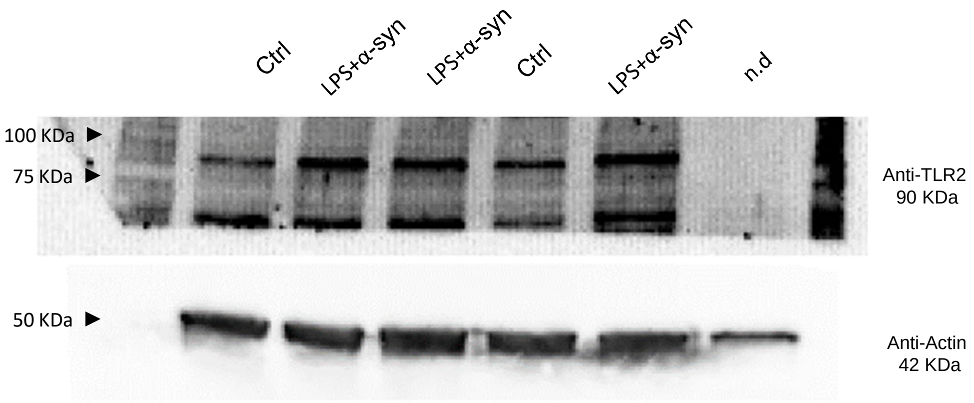

Caco2 cell

B

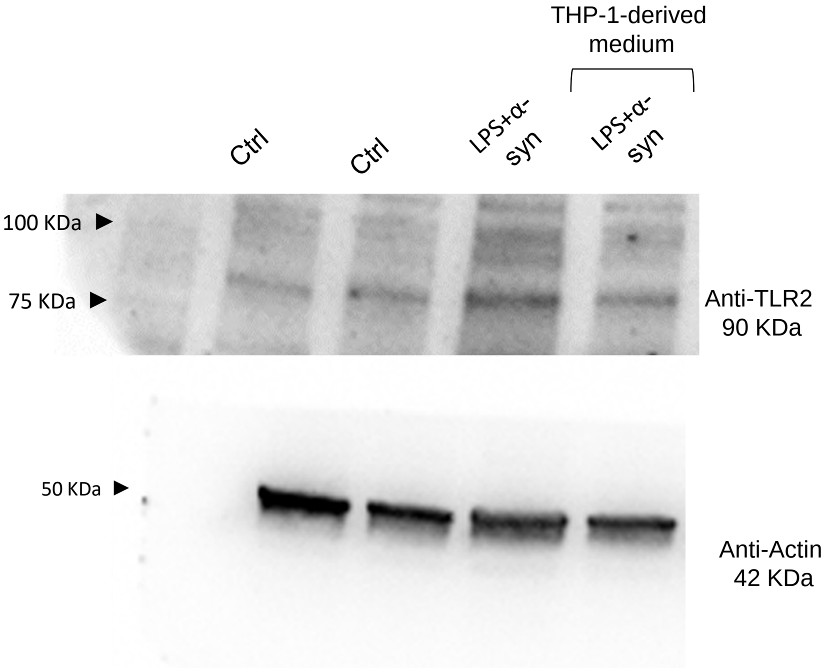

Representative "uncropped" blots of TLR-2 expression assessed by Western blot assay in cultured THP-1 (**A**) and Caco-2 (**B**) cells treated with LPS and α-syn and Caco-2 incubated with conditioned medium derived from THP-1 cells treated with α-syn and LPS. These blots correspond to Figure 5C in the manuscript
