# Supplementary material for: Enteric α-synuclein impairs intestinal epithelial barrier through caspase-1-inflammasome signaling in Parkinson’s disease before brain pathology
Source: NPJ Parkinsons Dis. 2022 Jan 12;8:9. doi: 10.1038/s41531-021-00263-x (PMC8755783; doi:10.1038/s41531-021-00263-x)
Supplement: Supplementary file 2 — REPORTING SUMMARY [file 41531_2021_263_MOESM2_ESM.pdf]

nature portfolio

Corresponding author(s): npj Parkinson' NPJPARKD-00719R

Last updated by author(s): Jun 28, 2021

Reporting Summary

Nature Portfolio wishes to improve the reproducibility of the work that we publish. This form provides structure for consistency and transparency in reporting. For further information on Nature Portfolio policies, see our Editorial Policies and the Editorial Policy Checklist.

Statistics

For all statistical analyses, confirm that the following items are present in the figure legend, table legend, main text, or Methods section.

n/a	Confirmed	
☐	☒	The exact sample size (n) for each experimental group/condition, given as a discrete number and unit of measurement
☐	☒	A statement on whether measurements were taken from distinct samples or whether the same sample was measured repeatedly
☐	☒	The statistical test(s) used AND whether they are one- or two-sided<br>*Only common tests should be described solely by name; describe more complex techniques in the Methods section.*
☒	☐	A description of all covariates tested
☐	☒	A description of any assumptions or corrections, such as tests of normality and adjustment for multiple comparisons
☐	☒	A full description of the statistical parameters including central tendency (e.g. means) or other basic estimates (e.g. regression coefficient) AND variation (e.g. standard deviation) or associated estimates of uncertainty (e.g. confidence intervals)
☐	☒	For null hypothesis testing, the test statistic (e.g. F, t, r) with confidence intervals, effect sizes, degrees of freedom and P value noted<br>*Give P values as exact values whenever suitable.*
☒	☐	For Bayesian analysis, information on the choice of priors and Markov chain Monte Carlo settings
☒	☐	For hierarchical and complex designs, identification of the appropriate level for tests and full reporting of outcomes
☒	☐	Estimates of effect sizes (e.g. Cohen's d, Pearson's r), indicating how they were calculated

Our web collection on statistics for biologists contains articles on many of the points above.

Software and code

Policy information about availability of computer code

Data collection	no software was used
Data analysis	GraphPad Prism, version 7.0 from GraphPad Software Inc., San Diego, CA, USA

For manuscripts utilizing custom algorithms or software that are central to the research but not yet described in published literature, software must be made available to editors and reviewers. We strongly encourage code deposition in a community repository (e.g. GitHub). See the Nature Portfolio guidelines for submitting code & software for further information.

Data

Policy information about availability of data

All manuscripts must include a data availability statement. This statement should provide the following information, where applicable:

- Accession codes, unique identifiers, or web links for publicly available datasets
- A description of any restrictions on data availability
- For clinical datasets or third party data, please ensure that the statement adheres to our policy

Data availability: The data that supports the findings of this study are available from the corresponding authors upon reasonable request.

Field-specific reporting

Please select the one below that is the best fit for your research. If you are not sure, read the appropriate sections before making your selection.

☒ Life sciences ☐ Behavioural & social sciences ☐ Ecological, evolutionary & environmental sciences

For a reference copy of the document with all sections, see nature.com/documents/nr-reporting-summary-flat.pdf

Life sciences study design

All studies must disclose on these points even when the disclosure is negative.

Sample size	To estimate the appropriate number of animals to include in each group, we conducted an "a priori" poweranalysis. The sample size estimation was conducted considering spinal cord cytochrome c oxidase activity. For this parameter, the expected difference between means in nTg and Tg groups is 0.05 with a SD of 0.02. Calculating the sample size using ANOVA, with a power of 0.8 and an α error of 0.05, we estimated that 5 animals for each group was an appropriate number to obtain a statistical significance.
Data exclusions	Outliers were defined as values that exceed the distance from the median value by 50%
Replication	The experimental procedures used for this study were performed with standardized methods to obtaine reproducible data. All data are reproducible
Randomization	This parameter is not relevant for this study since we used two different strains (trangenic mice A53T and their control littermates)
Blinding	The investigators were blinded to group allocation during data analysis

Reporting for specific materials, systems and methods

We require information from authors about some types of materials, experimental systems and methods used in many studies. Here, indicate whether each material, system or method listed is relevant to your study. If you are not sure if a list item applies to your research, read the appropriate section before selecting a response.

Materials & experimental systems

n/a	Involved in the study	
☐	☒	Antibodies
☐	☒	Eukaryotic cell lines
☒	☐	Palaeontology and archaeology
☐	☒	Animals and other organisms
☒	☐	Human research participants
☒	☐	Clinical data
☒	☐	Dual use research of concern

Methods

n/a	Involved in the study	
☒	☐	ChIP-seq
☒	☐	Flow cytometry
☒	☐	MRI-based neuroimaging

Antibodies

Antibodies used	GFAP (ab7260, Abcam, lot n: GR3215893-1), β-actin (ab8227, Abcam, lot n: no longer available), zonulin-1 (ab96587, Abcam, lot n: GR315642-34), occludin (ab31721, Abcam, lot n: GR119583-69), TLR-2 (sc-21759, Santa Cruz, Dallas, TX, USA, lot n: I2515). Secondary antibody: anti-mouse (ab97040 Abcam, lot n: GR3219575-7) and anti-rabbit (ab6721, Abcam, lot n: GR3381916-2)
Validation	In this study, we used the recommendations for the dilution reported in the manufacturer's datasheet. Datasheet links: https://www.abcam.com/gfap-antibody-ab7260.html; https://www.abcam.com/beta-actin-antibody-ab8227.html; https://www.abcam.com/zo1-tight-junction-protein-antibody-ab96587.html; https://www.abcam.com/occludin-antibody-ab31721.html; https://www.abcam.com/goat-mouse-igg-hl-hrp-preadsorbed-ab97040.html; https://www.abcam.com/goat-rabbit-igg-hl-hrp-ab6721.html; https://www.scbt.com/p/tlr2-antibody-tl2-1

Eukaryotic cell lines

Policy information about cell lines

Cell line source(s)	THP-1 cells were donated by Prof. Veit Hornung (Ludwig Maximilian University of Munich). The cell line CaCo2- were donated by Prof. Giudo Bocci (Department of Clinical and Experimental Medicine, University of Pisa, Italy)
Authentication	The authentification number of cell lines was previously provided to Prof. Veit Hornung (Ludwig Maximilian University of Munich) and Prof. Giudo Bocci (Department of Clinical and Experimental Medicine, University of Pisa, Italy)

Mycoplasma contamination	cell lines were tested monthly for mycoplasma by MycoFluor™ Mycoplasma Detection Kit (Thermo Fisher). Cell lines used for this study were negative to mycoplasma contamination
Commonly misidentified lines (See ICLAC register)	n.a.

Animals and other organisms

Policy information about studies involving animals; ARRIVE guidelines recommended for reporting animal research

Laboratory animals	Transgenic mice: mice expressing the human A53T mutant of α-syn. their control littermates (non transgenic mice). Age: 3, 6 and 9 months of age. Sex: male
Wild animals	this study did not involve wild animals
Field-collected samples	this study did not involve samples collected from the field
Ethics oversight	All experiments with mice were performed according to the national and international laws for laboratory animal welfare and experimentation (EU directive n. 2010/63/EU and Italian DL n. 26/2014. Authorization protocol n. 213/2020-PR)

Note that full information on the approval of the study protocol must also be provided in the manuscript.
